# High‐Resolution LC–MS/MS Characterization of Budmunchiamine‐Type Alkaloids in *Albizia niopoides*

**DOI:** 10.1002/rcm.70142

**Published:** 2026-07-25

**Authors:** Maria L. A. Majevski, Lienne D'Auria Lima, Adriana C. C. Reis, Leonan I. C. R. Santos, Brenno F. S. Vargas, Markus Kohlhoff, Geraldo Célio Brandão

**Affiliations:** ^1^ Escola de Farmácia Universidade Federal de Ouro Preto, Campus Morro do Cruzeiro Ouro Preto Minas Gerais Brazil; ^2^ Laboratório de Química de Produtos Naturais Bioativos, Fundação Oswaldo Cruz Instituto René Rachou Belo Horizonte Minas Gerais Brazil

**Keywords:** *Albizia niopoides*, budmunchiamines, high‐resolution mass spectrometry, macrocyclic spermine alkaloids, natural product dereplication

## Abstract

**Rationale:**

Budmunchiamines are macrocyclic spermine alkaloids that constitute a distinctive class of polyamine natural products predominantly found in species of the genus *Albizia*. Comprehensive characterization of these compounds remains challenging because of their structural diversity and the limited availability of reference standards. This study aimed to profile the budmunchiamine alkaloids present in the trunk bark of *Albizia niopoides* using high‐resolution tandem mass spectrometry.

**Methods:**

Alkaloids were extracted from the trunk bark of *A. niopoides* and analyzed by ultrahigh‐performance liquid chromatography coupled with electrospray ionization quadrupole time‐of‐flight mass spectrometry (UHPLC–ESI–QTOF–MS) operated in positive ionization mode. Compound annotation was based on accurate mass measurements (≤ 5 ppm), chromatographic retention behavior, isotopic patterns, and diagnostic MS/MS fragmentation, supported by comparison with published data.

**Results:**

Twenty‐six macrocyclic spermine alkaloids were detected and annotated. The MS/MS spectra consistently exhibited characteristic budmunchiamine fragment ions at *m/z* 214, 171, 169, and 129, together with diagnostic neutral losses, confirming assignment to this structural class. Six compounds were identified as the known budmunchiamines L1, L4, L5, and L6, whereas two corresponded to previously synthesized analogues (Compounds 2 and 21). The remaining 20 compounds displayed distinct precursor ions but closely related fragmentation pathways, indicating previously unreported budmunchiamine‐type analogues.

**Conclusions:**

UHPLC–ESI–QTOF–MS/MS proved to be an effective strategy for the dereplication and structural annotation of macrocyclic spermine alkaloids from *A. niopoides*. The results substantially expand the known chemical diversity of budmunchiamines in this species and demonstrate the value of high‐resolution tandem mass spectrometry for the characterization of structurally complex natural products.

## 1 Introduction

Advances in instrumental ultrahigh‐performance liquid chromatography (UHPLC) and high‐resolution mass spectrometry (HRMS) have significantly transformed the analysis of natural products by enabling the rapid detection and structural characterization of chemically complex metabolites directly from crude extracts. In particular, electrospray ionization coupled to quadrupole time‐of‐flight mass spectrometry (ESI–QTOF–MS) provides accurate mass measurements, high resolving power, and information rich tandem mass spectrometric data that are especially valuable for nitrogen‐containing secondary metabolites, including polyamine derived alkaloids [1, 2]. These capabilities are critical for compound classes that display high structural similarity, extensive homologous series, and subtle variations in substitution patterns. Recent advances in UHPLC–HRMS applications for natural products and quality control illustrate the technique's broad utility for structural elucidation and fingerprinting of plant metabolites [3].

The genus *Albizia* (Fabaceae, Mimosoideae) is chemically diverse and has been extensively investigated for its ethnopharmacological relevance. Previous phytochemical studies have reported saponins, flavonoids, tannins, triterpenoids, phenolic glycosides, and alkaloids across multiple species [4]. Such chemical diversity has been associated with the broad spectrum of biological activities reported for *Albizia* species and highlights the genus as a valuable source of bioactive natural products [4, 5, 6].

Among these metabolites identified in *Albizia*, macrocyclic spermine alkaloids commonly known as budmunchiamines constitute a chemically distinctive group characterized by a spermine‐derived macrocyclic core acylated with long‐chain fatty acids. To date, at least 40 structurally characterized macrocyclic spermine alkaloids have been reported [5, 6, 7, 8, 9, 10, 11, 12, 13, 14], including 25 distinct budmunchiamine derivatives identified within the genus *Albizia* [5, 6, 9, 11, 12, 13, 14, 15]. These comprise budmunchiamines A–I (from *Albizia amara* ) [5, 6, 9], L1–L6 (from *A. lebbeck* and *Albizia schimperiana* ) [9, 11], and various *N*‐demethylated and hydroxylated derivatives [12, 16, 17]. Notably, four putative novel budmunchiamine derivatives were tentatively characterized in *Albizia lucidior* [14]. These compounds appear to be structurally related to budmunchiamines B, C, G, and K, differing primarily in the presence of propene or ethylene substituents. The occurrence of these alkaloids is consistent with previous phytochemical investigations demonstrating that macrocyclic spermine alkaloids are characteristic constituents of several *Albizia* species, including *Albizia gummifera* , *A. schimperiana* , *A. amara* , *A. lebbeck* , *A. lucidior*, *A. procera* , *A. guachapele*, *A. anthelmintica*, and *Albizia adinocephala* [5, 6, 9, 11, 12, 14, 15, 16, 18].

Beyond their chemotaxonomic significance, budmunchiamines have attracted considerable scientific interest owing to their diverse biological properties, including antibacterial, antifungal, antioxidant, cytotoxic, anticholinesterase, and antiplasmodial activities. The antiplasmodial activity of budmunchiamines against *Plasmodium falciparum* has been particularly well documented, with structure–activity relationship studies demonstrating that activity is strongly dependent on the length and oxidation state of the fatty acyl substituent [6, 17]. Additionally, recent studies have revealed promising anticholinesterase activity of budmunchiamine alkaloids, highlighting their potential in the treatment of Alzheimer's disease [19].

*Albizia niopoides* (Benth.) Burkart., popularly known in Brazil as farinha‐seca, is widely used in traditional medicine. Ethnobotanical reports for species of this genus describe the use of stem bark decoctions to treat fever, respiratory and gastrointestinal disorders, and infectious diseases [20, 21, 22]. Despite its traditional relevance, the chemical composition of *A. niopoides* remains poorly characterized when compared to other members of the genus. Existing studies have largely focused on general metabolite classes, with limited information on individual alkaloid constituents [23]. Given the structural complexity of budmunchiamines and the challenges associated with their isolation, HRMS‐based strategies provide an efficient alternative for their detection and structural annotation.

The structural complexity of budmunchiamine‐type alkaloids, characterized by large macrocyclic polyamine frameworks, multiple *N*‐methylation patterns, and variable side chain oxidation, poses significant challenges for classical phytochemical approaches alone. In this context, UHPLC and ESI–QTOF–HRMS, combined with tandem MS experiments, offers decisive advantages by providing sub‐ppm mass accuracy, rich fragmentation data, and high confidence molecular formula assignments, facilitating both dereplication and structural annotation of known and potentially novel analogues [24, 25, 26].

In the present study, we employed ESI–QTOF–HRMS and MS/MS analyses to investigate the alkaloid profile of the trunk bark of *Albizia niopoides*. This mass spectrometry centered approach enabled the identification and structural characterization of budmunchiamine‐type alkaloids, including previously unreported analogues. The results expand the chemical knowledge of *A. niopoides* and further demonstrate the effectiveness of quadrupole time‐of‐flight mass spectrometry for the detailed analysis of complex polyamine alkaloids in plant derived matrices.

## 2 Materials and Methods

### 2.1 Plant Material

Stem bark of Albizia niopoides (Benth.) Burkart. was collected in Santana de Pirapama, Minas Gerais, Brazil (19°00′21″ S, 44°02′34″ W). Botanical identification was carried out by Prof. João Renato Stehmann (Institute of Biological Sciences, Federal University of Minas Gerais). A voucher specimen (OUPR 33503) was deposited at the Professor José Badini Herbarium (Federal University of Ouro Preto).

### 2.2 Extraction

The plant material was air dried at temperatures below 40°C and milled to a homogeneous powder. Exhaustive extraction was performed by cold percolation using ethanol at room temperature. The extract was filtered and concentrated under reduced pressure at 45°C, followed by drying at 50°C to constant weight.

### 2.3 Sample Preparation

Aliquots of the dried extract (2.5 mg) were dissolved in HPLC‐grade methanol (1.0 mL), sonicated, and centrifuged (10 000 rpm, 5 min). The supernatant was filtered through a 0.22‐μm PVDF membrane prior to analysis.

### 2.4 UHPLC–HRMS/MS Analysis

UHPLC–HRMS/MS analyses were performed using a Shimadzu Nexera UHPLC system hyphenated to a Bruker maXis ESI–QTOF mass spectrometer operating in positive‐ion mode. Chromatographic separation was achieved on a C18 column (2.0 × 150 mm, 2.2 μm) at 40°C using water (A) and acetonitrile (B), both containing 0.1% formic acid. The flow rate was 0.4 mL min^−1^ with a gradient from 5% to 100% B over 11 min.

Mass spectra were acquired over *m/z* 100–1500 at 5 Hz. Data‐dependent MS/MS experiments were conducted using collision‐induced dissociation (CID) with collision energies between 15 and 60 eV. External calibration was performed using sodium formate, and postacquisition recalibration was applied. Molecular formula assignment and tentative identification were based on accurate mass, isotopic distribution, and MS/MS comparison with literature data and public databases [26, 27, 28, 29].

## 3 Results and Discussion

UHPLC–ESI–QTOF–HRMS analysis of the ethanolic trunk bark extract of *Albizia niopoides* revealed a chemically homogeneous yet structurally diverse profile of nitrogen rich constituents detected exclusively in positive ionization mode. A total of 26 chromatographic peaks were assigned to macrocyclic spermine‐based alkaloids on the basis of accurate mass measurements (≤ 5 ppm), isotopic patterns, chromatographic behavior, and tandem mass spectrometric data (Table 1). This alkaloid profile is fully consistent with the established chemical space of budmunchiamine‐type macrocyclic spermine alkaloids reported for the genus *Albizia* [9, 11, 12, 16, 18], which typically occur as homologous series of structurally related metabolites rather than as isolated compounds.

**TABLE 1:** UHPLC–ESI–QTOF–HRMS/MS data for budmunchiamine‐type alkaloids detected in the ethanolic trunk bark extract of *Albizia niopoides*.

	Compounds	Area %	Molecular formula	RT (min)	UV	Characteristic m/z of ions in positive ion mode (%)	HRMS [M + H]^+^ (*m/z*)	Error (ppm)
**1**	8‐(11‐hydroxyundecyl)‐1,5,9,13‐tetraazacycloheptadecan‐6‐one new budmunchiamine‐type alkaloid structure	3.02	C_24_H_50_N_4_O_2_	1.1	190, 209	427.3999 (100.0), 353.3142 (6.0), 282.2430 (8.8), 214.2161 (13.3), 214.1901 (9.9), 196.2067 (11.7), 171.1493 (25.1), 169.1338 (28.7), 129.1388 (7.7)	427.4001	2.6
**2**	8‐(8‐Hydroxyundecy1)‐1,5,9,13‐tetraazacycloheptadecan Budmunchiamine‐type alkaloid structure*	2.1	C_24_H_50_N_4_O_2_	3.2	190, 208	427.3996 (100.0), 353.3180 (7.2), 282.2417 (5.4), 214.2168 (13.7), 214.1910 (9.7), 196.2053 (8.2), 171.1495 (26.1), 169.1335 (31.9), 129.1381 (12.1)	427.3998	3.3
**3**	8‐(6,7‐Dihydroxypentadencyl)‐1,5,9,13‐tetraazacycloheptadecan new budmunchiaminetype alkaloid structure	8.69	C_28_H_58_N_4_O_3_	3.7	190, 209	499.4574 (100.0), 425.3738 (6.1), 268.2636 (8.6), 250.2524 (7.2), 214.1918 (10.5), 171.1491 (21.8), 169.1336 (31.2), 129.1385 (7.5)	499.4571	3.2
**4**	8‐(10‐Hydroxytridency1)‐1,5,9,13‐tetraazacycloheptadecan new budmunchiamine‐type alkaloid structure	73.37	C_26_H_54_N_4_O_2_	3.8	190, 209	437.4211 (47.8), 420.3949 (15.1), 395.3739 (61.1), 381.3582 (100.0), 367.3426 (72.7), 353.3271 (35.0), 339.3111 (16.4), 224.2369 (12,6), 171.1492 (22.5), 169.1336 (23.4)	455.4316	3.6
**5**	8‐(6,7‐Dihydroxypentadencyl)‐1,5,9,13‐tetraazacycloheptadecan new budmunchiamine‐type alkaloid structure	9.65	C_30_H_62_N_4_O_3_	4.1	190, 210	527.4888 (100.0), 296.2949 (11.6), 214.1903 (10.6), 171.1489 (25.3), 169.1336 (41.7), 129.1385 (11.1)	527.4886	1.9
**6**	8‐Undecyl‐1,5,9,13‐tetraazacycloheptadecan‐6‐one new budmunchiamine‐type alkaloid structure	57.72	C_24_H_50_N_4_O	4.2	190, 211	411.4055 (82.0), 394.3791 (100.0), 337.3209 (22.6), 266.2474 (25.1), 243.2792 (28.8), 238.2527 (31.8), 224.2373 (53.3), 198.2219 (89.7), 184.2061 (32.1), 171.1494 (25.1)	411.4057	1.2
**7**	8‐(15‐Hydroxypentadencyl)‐1,5,9,13‐tetraazacycloheptadecan‐6‐one new budmunchiamine‐type alkaloid structure	3.76	C_28_H_58_N_4_O_2_	4.3	190, 210	465.4526 (35.9), 423.4056 (44.5), 409.3900 (100.0), 395.3741 (95.9), 381.3586 (74.5), 367.3429 (55.4), 353.3270 (30.7), 339.3115 (12.6), 171.1493 (19.9), 169.1336 (22.3)	483.4629	2.1
**8**	8‐(10, 11‐Dihydroxynonadencyl)‐1,5,9,13‐tetraazacycloheptadecan‐6‐one new budmunchiamine‐type alkaloid structure	4.99	C_32_H_66_N_4_O_3_	4.4	190, 212	555.5197 (100.0), 324.3254 (9.5), 306.3145 (6.5), 214.1912 (8.6), 171.1493 (24.3), 169.1338 (31.0), 129.1385 (7.5)	555.5202	2.0
**9**	8‐Dodecyl‐1,5,9,13‐tetraazacycloheptadecan‐6‐one new budmunchiamine‐type alkaloid structure	5.91	C_25_H_52_N_4_O	4.5	190, 212	425.4209 (100.0), 214.1911 (8.3), 171.1495 (41.7), 169.1343 (40.9), 129.1391 (17.7)	425.4207	2.8
**10**	8‐Tridecyl‐1,5,9,13‐tetraazacycloheptadecan‐6‐one budmunchiamine L4	72.76	C_26_H_54_N_4_O	4.7	190, 212	439.4368 (100.0), 422.4103 (81.2), 323.3417 (13.8), 271.3104 (13.9), 266.2839 (13.0), 252.2683 (22.2), 226.2530 (42.5), 214.1915 (10.1), 212.2373 (12.2)	439.4366	2.0
**11**	8‐((1E,3Z,12E,15Z,18E,20E)‐6,17,22‐trihydroxydocosa‐1,3,12,15,18,20‐hexaen‐1‐yl)‐1,5,9,13‐tetraazacycloheptadecan‐6‐one New budmunchiamine‐type alkaloid structure	4.85	C_35_H_60_N_4_O_4_	4.9	190, 212	601.467 (100.0), 214.1917 (15.7), 171.1490 (37.4), 169.1337 (61.7), 165.0554 (15.2), 147.0436 (21.1), 129.1386 (16.3)	601.4679	2.2
**12**	(E)‐8‐(pentadec‐11‐en‐1‐yl)‐1,5,9,13‐tetraazacycloheptadecan‐6‐one budmunchiamine L6	9.32	C_28_H_56_N_4_O	5.0	190, 212	465.4520 (100.0), 391.3677 (6.6), 252.2686 (12.7), 214.1918 (6.2), 171.1491 (21.3), 169.1336 (25.7), 129.1387 (6.7)	465.4524	0.4
**13**	8‐(15Hydroxnonadecyl)‐1,5,9,13‐tetraazacycloheptadecan‐6‐one new budmunchiamine‐type alkaloid structure	28.10	C_32_H_66_N_4_O_2_	5.1	190, 212	521.5150 (40.5), 465.4528 (36.1), 451.4367 (60.2), 437.4205 (71.5), 423.4054 (87.5), 409.3899 (100.0), 395.3742 (86.8), 381.3582 (63.1), 367.3430 (36.3), 169.1336 (25.9)	539.5255	1.1
**14**	8‐Pentadencyl‐1,5,9,13‐tetraazacycloheptadecan‐6‐one budmunchiamine L5	100.0	C_28_H_58_N_4_O	5.2	190, 212	467.4683 (100.0), 450.4418 (45.7), 351.3730 (6.2), 294.3157 (5.2), 280.2997 (8.2), 254.2843 (17.8), 240.2686 (5.4), 214.1916 (5.0), 171.1491 (4.3)	467.4681	1.5
**15**	(E)‐8‐(dodeca‐7,11‐dien‐l‐yl)‐1,5,9,13 tetraazacycloheptadecan‐6‐one new budmunchiamine‐type alkaloid structure	8.23	C_25_H_48_N_4_O	5.3	190, 212	421.3902 (100.0), 350.3161 (24.4), 266.2478 (39.7), 251.2482 (34.0), 224.2373 (27.1), 209.1884 (4.1), 203.2234 (11.1)	421.3894	2.8
**16**	8‐hexadecyl‐1,5,9,13‐tetraazacycloheptadecan‐6‐one budmunchiamine L1	8.03	C_29_H_60_N_4_O	5.5	190, 212	481.4832 (100.0), 439.4370 (11.1), 268.3004 (13.4), 214.1917 (9.5), 171.1492 (28.10), 169.1336 (35.7), 129.1386 (8.7)	481.4834	2.3
**17**	8‐Heptadecyl‐1,5,9,13‐tetraazacycloheptadecan‐6‐one new budmunchiamine‐type alkaloid structure	73.87	C_30_H_62_N_4_O	5.6	190, 212	495.4993 (100.0), 478.4729 (32.0), 379.4043 (5.6), 327.3730 (3.7), 322.3465 (4.0), 308.3309 (6.4), 282.3153 (13.2), 268.2997 (3.5), 214.1911 (4.9), 171.1493 (3.0)	495.4992	1.8
**18**	8‐((1E,3Z)‐dodeca‐1,3‐dien‐l‐yl)‐1,5,9,13‐tetraazacycloheptadecan‐6‐one new budmunchiamine‐type alkaloid structure	7.4	C_27_H_52_N_4_O	5.8	190, 213	449.4212 (100.0), 378.3473 (12.8), 294.2790 (20.8), 279.2798 (20.9), 252.2685 (21.5), 250.2527 (3.1)	449.4214	1.1
**19**	8‐Octadencyl‐1,5,9,13‐tetraazacycloheptadecan‐6‐one new budmunchiamine‐type alkaloid structure	10.49	C_31_H_64_N_4_O	5.9	190, 213	509.5141 (100.0), 435.4300 (5.9), 364.3571 (5.3), 296.3309 (11.0), 214.1911 (7.1), 171.1492 (20.7), 169.1336 (27.7), 129.1384 (5.6)	509.5147	2.1
**20**	8‐Nonadecyl‐1,5,9,13‐tetraazacycloheptadecan‐6‐one new budmunchiamine‐type alkaloid structure	96.3	C_32_H_66_N_4_O	6.0	190, 214	523.5304 (100.0), 506.5041 (17.7), 407.4354 (3.2), 336.3619 (2.8), 310.3465 (6.7), 214.1914 (2.8)	523.5304	1.9
**21**	8‐Henicosyl‐1,5,9,13‐tetraazacycloheptadecan‐6‐one budmunchiamine‐type alkaloid structure*	8.97	C_34_H_70_N_4_O	6.4	190, 215	551.5611 (100.0), 477.4764 (5.7), 406.4042 (5.2), 338.3779 (9.4), 214.1911 (7.5), 171.1492 (23.7), 169.1336 (32.2), 129.1387 (5.8)	551.5613	2.5
**22**	(E)‐8‐(21,22‐dihydroxydocos‐19‐en‐l‐yl)‐1,5,9,13‐tetraazacycloheptadecan‐6‐one new budmunchiamine‐type alkaloid structure	4.53	C_35_H_70_N_4_O_3_	6.5	190, 215	595.5515 (100.0), 551.5627 (8.4), 466.4710 (9.1), 378.3723 (20.6), 350.3776 (17.6), 336.3617 (38.6), 310.3464 (40.7), 171.1491 (30.3), 169.1336 (24.4)	595.5517	1.5
**23**	8‐((5E,10E)hexadeca‐5,10‐dien‐l‐yl)‐1,5,9,13‐tetraazacycloheptadecan‐6‐one new budmunchiamine‐type alkaloid structure	2.39	C_29_H_56_N_4_O	6.6	190, 215	477.4518 (100.0), 394.3867 (10.4), 393.3833 (33.6), 323.3140 (15.4), 322.3101 (65.7), 265.2527 (3.0), 221.1763 (3.3)	477.4521	2.3
**24**	8‐((9E,10Z)‐octadeca‐9,15‐dien‐l‐yl)‐1,5,9,13‐tetraazacycloheptadecan‐6‐one new budmunchiamine‐type alkaloid structure	11.20	C_31_H_60_N_4_O	6.7	190, 220	505.4835 (100.0), 434.4098 (4.6), 350.3408 (7.3), 309.3342 (3.3), 156.1497 (2.4)	505.4835	2.0
**25**	8‐((2E,15Z)‐icosa‐2,15‐dien‐l‐yl)‐1,5,9,13‐tetraazacycloheptadecan‐6‐one new budmunchiamine‐type alkaloid structure	11.83	C_33_H_65_N_4_O	7.1	190, 217	429.4225 (0.8), 378.3830 (6.2), 321.3253 (8.0), 303.2849 (7.3), 214.1906, 213.1976 (100.0), 156.1396 (8.2), 149.1329 (2.4), 135.1168 (4.8), 129.1388 (27.7)	533.5149	1.7
**26**	8‐((1E,3Z,8E)‐6‐hydroxyhenicosa‐1,3,8‐trien‐l‐yl)‐1,5,9,13‐tetraazacycloheptadecan‐6‐one New budmunchiamine‐type alkaloid structure	0.78	C_34_H_64_N_4_O_2_	8.9	190, 219	561.5091 (100.0), 379.3784 (1.0), 378.3744 (3.0), 140.1070 (1.0), 139.1234 (1.4)	561.5095	2.1

*Note:* Retention time (RT), relative peak area (%), molecular formula, UV absorption maxima, accurate mass of the protonated molecule ([M + H]^+^), mass error (ppm), * compounds had already been obtained by synthesis [16, 30] and major product ions observed in positive‐ion CID MS/MS experiments are shown. Molecular formulas were assigned based on accurate mass measurements (≤ 5 ppm) and isotopic distribution. Fragment ions are listed as *m/z* values with relative intensities (%) in parentheses.

All compounds were detected as protonated molecules ([M + H]^+^), with precursor ions spanning an *m/z* range from 411.4057 to 601.4679 and eluting between 1.1 and 8.9 min under the applied chromatographic conditions (Figure 1). The alkaloid distribution was dominated by a limited number of high abundance constituents, whereas the remaining compounds were present as lower intensity homologues, a pattern characteristic of budmunchiamine producing *Albizia* species [9, 11, 12, 16, 18]. Accurate masses, retention times, elemental compositions, and diagnostic fragment ions are summarized in Table 1 and complete spectrometric data with representative fragmentation pathways are provided in Figures S1–S101. Representative structures of macrocyclic spermine‐based alkaloids are shown in Figure 2.

**FIGURE 1:**
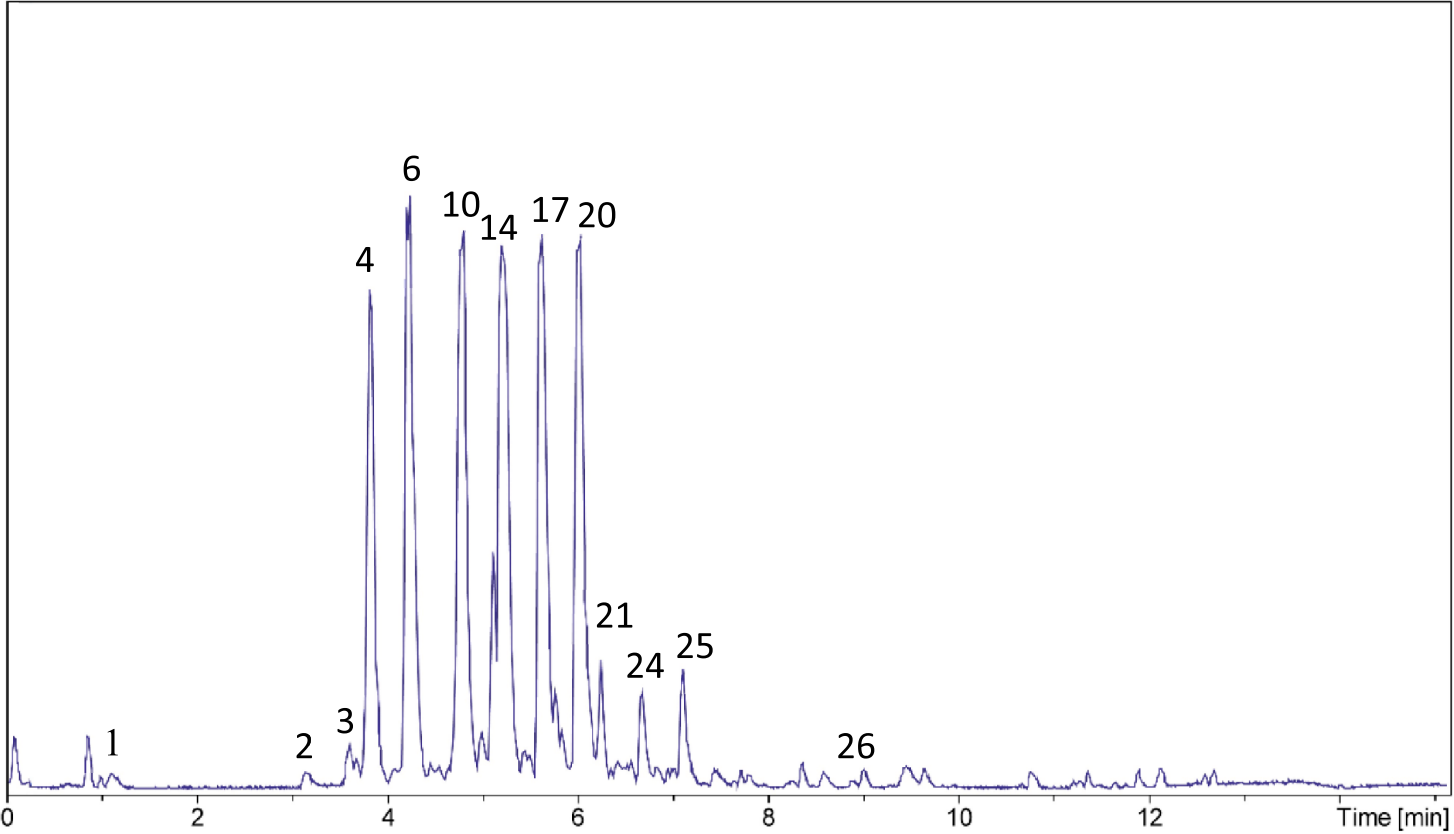
RP‐UPLC profile of ethanolic extract of *A. niopoides* trunk. Conditions: C18 column (2.0 × 150 mm, 2.2 μm). Elution was carried out with a linear gradient of water with 0.1% formic acid and acetonitrile with 0.1% formic acid (from 5% to 100% of acetonitrile with 0.1% formic acid in 11 min), and the UPLC fingerprints were registered on a Nexera UHPLC system (Shimadzu, Kyoto, Japan).

**FIGURE 2:**
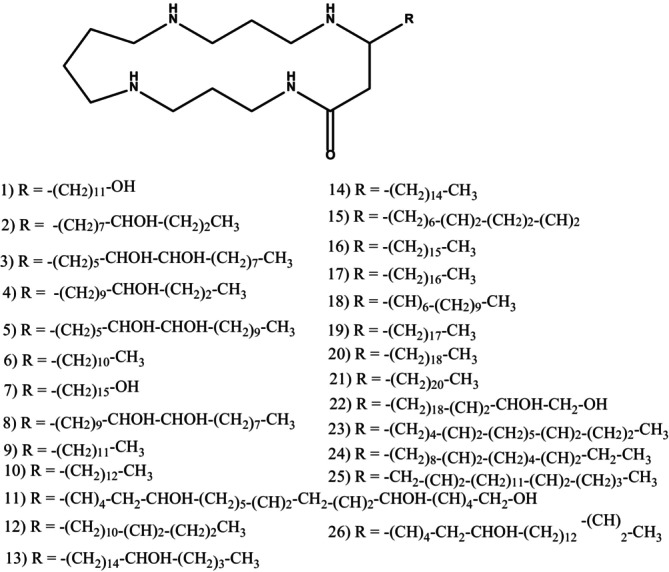
Representative structures of macrocyclic spermine based alkaloids of *Albizia niopoides*.

Across the dataset, CID MS/MS spectra displayed highly conserved fragmentation behavior typical of macrocyclic spermine alkaloids. Product ions at *m/z* 214, 171, 169, and 129 were observed for the majority of compounds and correspond to hallmark fragments arising from charge stabilized cleavages of the polyamine backbone. These diagnostic ions have been consistently reported for budmunchiamines isolated from other *Albizia* species [9, 11, 12, 16] and were detected irrespective of precursor *m/z*, retention time, or degree of substitution. A frequent neutral loss of 74 Da, commonly attributed to elimination of a 1,3‐propanediamine unit from the spermine framework, further supports the assignment of all detected compounds to the budmunchiamine structural class [9, 11, 16].

The structural annotation of the 20 new budmunchiamine‐type alkaloids detected in this study was performed through a comprehensive analysis of accurate mass measurements, isotopic patterns, characteristic MS/MS fragmentation pathways, and chromatographic behavior, in conjunction with comparison to literature data for known budmunchiamines. It is important to emphasize that, due to the inherent limitations of MS‐based characterization, the exact positions of hydroxyl groups and double bonds on the fatty acyl side chains (R) could not be unambiguously determined without isolation and NMR spectroscopic analysis [19, 31, 32]. The assignments presented herein represent the most plausible structural interpretations based on the available mass spectrometric data, especially fragments of the fatty acyl side chains formed in the MS^2^ analyses.

For hydroxylated analogues, the positions of OH substituents on the acyl side chain were tentatively assigned based on the following criteria: (i) the molecular formula indicating the presence of one or more oxygen atoms in excess of the amide carbonyl; (ii) the retention time, where hydroxylated compounds generally elute earlier than their nonhydroxylated counterparts of similar molecular weight due to increased polarity; (iii) the presence of characteristic MS/MS fragment ions attributable to cleavage adjacent to the hydroxylated carbon; and (iv) comparison with previously reported hydroxylated budmunchiamines in the literature [19, 31, 32, 33]. For example, Compounds 1 and 2, both with the molecular formula C_2_₄H₅₀N₄O_2_ (*m/z* 427.4001 and 427.3998, respectively), represent isomeric hydroxylated budmunchiamine analogues. Their distinct retention times (1.1 and 3.2 min) and subtle differences in relative fragment ion intensities support their annotation as structural isomers differing in the position and/or stereochemistry of the hydroxyl substituents on the acyl chain. However, the exact positions of the OH groups (e.g., at C‐8′, C‐11′, or other positions) cannot be definitively assigned based solely on MS/MS data. In Figure 2, the OH position for Compound 2 is tentatively indicated at C‐8′ of the acyl chain, consistent with biosynthetic hydroxylation patterns observed in related *Albizia* species [10], whereas in Figure S4, an alternative position at C‐11′ is illustrated for Compound 1. Both representations are plausible, and definitive assignment would require isolation and 2D NMR experiments to establish correlations between the hydroxymethine proton and adjacent methylene groups [6, 16, 19].

**FIGURE 3:**
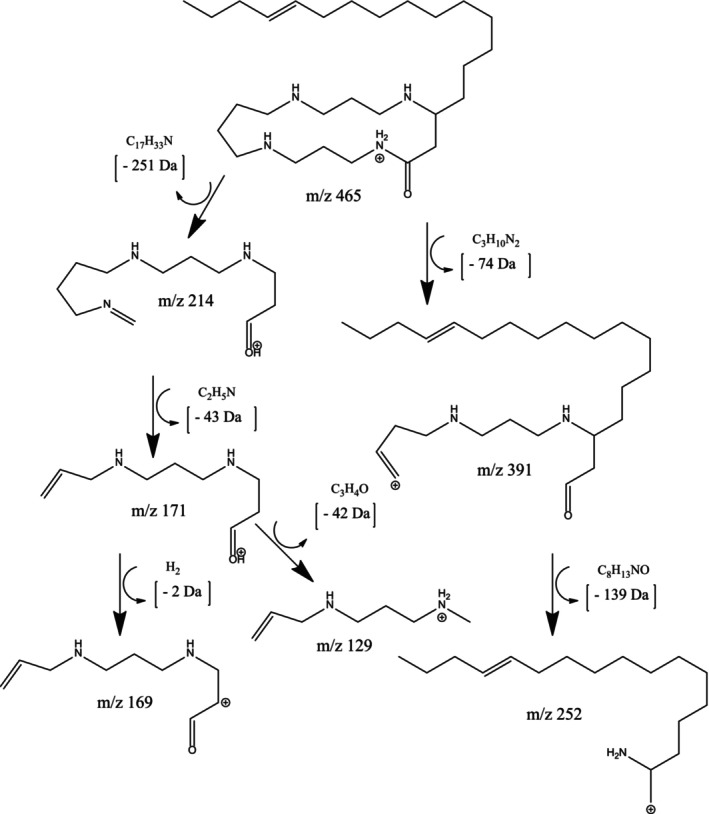
Fragmentation pathways of budmunchiamine L6 (Compound 8).

For unsaturated analogues, the presence and degree of unsaturation were inferred from the molecular formula (degree of unsaturation = number of rings and double bonds) and confirmed by the observation of characteristic MS/MS fragmentation patterns distinct from those of saturated analogues. Compound 23 (*m/z* 477.4521, C_2_₉H₅₆N₄O) exhibited a distinct fragmentation profile with prominent ions at *m/z* 394, 393, 323, and 322, consistent with the presence of a double bond in the acyl side chain. The reduced relative intensities of typical low‐mass polyamine fragments (*m/z* 214, 171, 169, 129) in Compound 23, compared to saturated analogues, suggest that unsaturation alters the gas phase dissociation pathways, possibly through charge localization effects or allylic cleavages [4, 11, 16]. However, the exact position of the double bond (e.g., Δ^2^′, Δ^6^′, or other positions) cannot be determined from MS/MS data alone. The fragmentation pathway shown in Figure 3 for Compound 23 represents a tentative assignment based on the most plausible cleavage patterns, but alternative positions remain possible. Previous studies on budmunchiamines from *A. lebbeck* and *A. amara* have reported unsaturated analogues with double bonds at various positions, and without NMR data, unambiguous localization is not feasible [6, 11, 18].

The 20 new alkaloids were named as “new budmunchiamine type alkaloid structure” followed by a compound number (Table 1), reflecting their tentative structural annotation. This nomenclature follows established conventions in natural products dereplication studies where MS‐based annotation precedes full structural characterization [24, 25]. The compounds are proposed as new budmunchiamine analogues based on (a) unique molecular formulas not previously reported in the literature; (b) characteristic budmunchiamine MS/MS fragmentation patterns; (c) logical mass differences (14, 28, or 42 Da) from known compounds corresponding to homologous series variations; and (d) chromatographic behavior consistent with the budmunchiamine class. The fragmentation schemes for all new compounds are provided in Figures S1–S101, enabling future researchers to compare and potentially confirm these assignments upon isolation.

Two early eluting peaks at 1.1 and 3.2 min displayed identical precursor ions at *m/z* 427.4001 and 427.3998 ([M + H]^+^, C_24_H_50_N_4_O_2_; Compounds 1 and 2), consistent with isomeric species. Although their elemental compositions were identical, their distinct retention times, and subtle differences in relative fragment ion intensities support their annotation as structural isomers. Such isomerism is frequently observed within budmunchiamine series and reflects biosynthetic variability in acyl substitution patterns [5, 16].

Several compounds formed clear homologous series differing by incremental mass increases attributable to variations in the fatty acyl substituent. Precursor ions at *m/z* 465.4524 (Compound 12), 481.4834 (Compound 16), 509.5147 (Compound 19), and 551.5613 (Compound 21) exhibited closely related MS/MS spectra dominated by canonical polyamine derived fragments together with higher mass ions retaining portions of the acyl residue. The ion at *m/z* 465.4524 matched the elemental composition and fragmentation behavior reported for budmunchiamine L6, enabling confident annotation [9, 11, 12]. Homologous series differing by 28‐Da increments reflect systematic changes in acyl chain length and represent a defining feature of budmunchiamines across the genus [9, 11, 12]. A representative fragmentation pathway for budmunchiamine L6 is shown in Figure 5.

Budmunchiamine L5 (Compound 14) was the most abundant constituent, accounting for 100% relative area within the normalized dataset. Its MS/MS spectrum showed a dominant precursor ion at *m/z* 467.4683 accompanied by fragment ions at *m/z* 450, 351, and 294 in addition to the characteristic low‐mass polyamine fragments. The predominance of one or two long‐chain budmunchiamines parallels observations in other *Albizia* species and suggests that acylation with long‐chain fatty acids is biosynthetically favored [4, 5, 6, 7, 9, 11, 12, 16].

**FIGURE 4:**
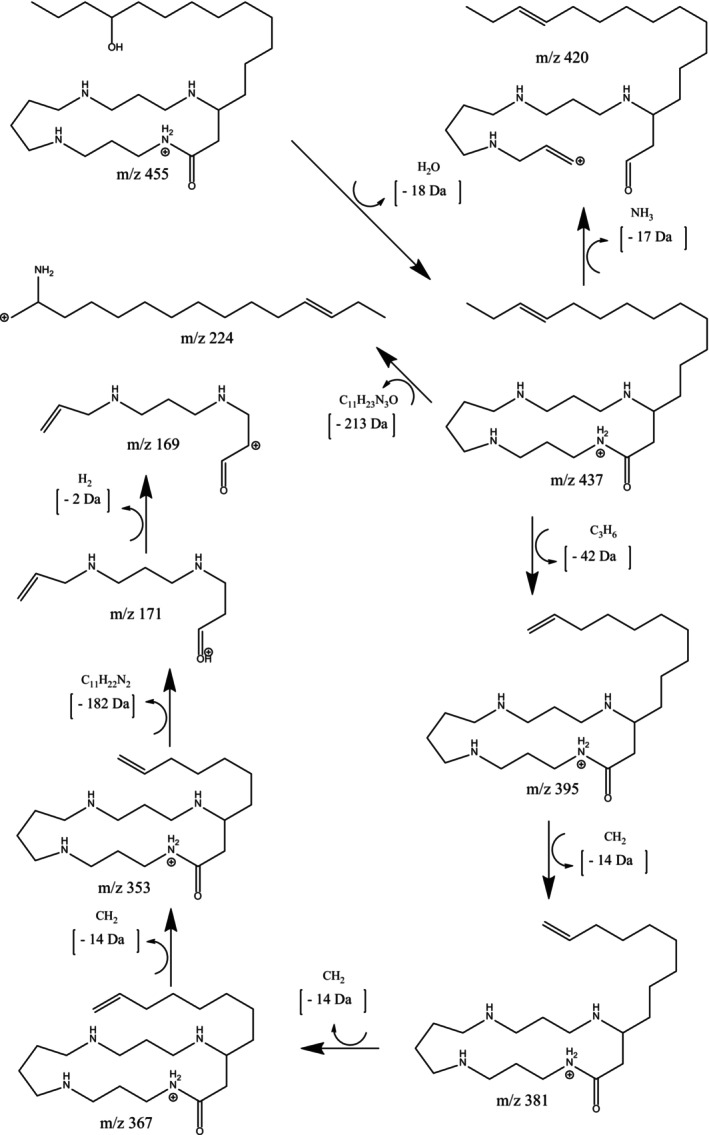
Fragmentation pathways of Compound 4.

Hydroxylated analogues constituted a significant portion of the alkaloid profile. Multiple compounds possessed oxygen‐rich molecular formulas while retaining the canonical spermine‐derived fragmentation pattern, indicating oxidative modification of the fatty acyl substituent without alteration of the macrocyclic core. Additional product ions attributable to neutral losses involving oxygenated moieties were detected, and representative fragmentation pathways are illustrated in Figure 4. The predominance of oxygenated analogues suggests that oxidative tailoring reactions play an important role in budmunchiamine biosynthesis in *A. niopoides* [16].

**FIGURE 5:**
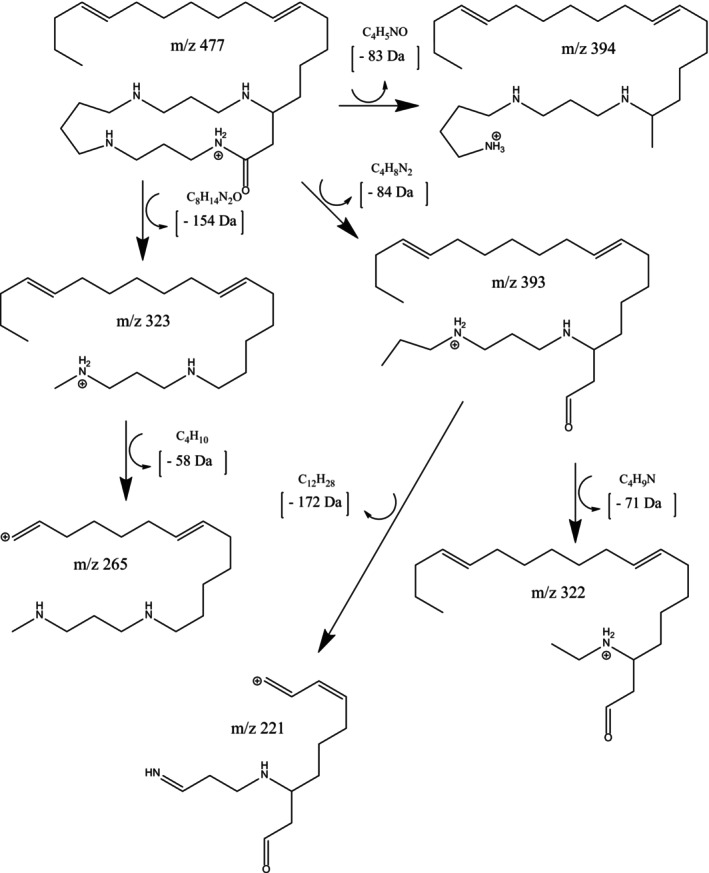
Fragmentation pathways of Compound 23.

Unsaturated acyl substituents were inferred for several compounds on the basis of accurate mass measurements and characteristic MS/MS behavior. In these cases, spectra showed conserved low‐mass polyamine fragments together with additional higher mass product ions not observed in saturated analogues. Compound 23 ([M + H]^+^, *m/z* 477.4521) displayed a distinct fragmentation profile with prominent ions at *m/z* 394, 393, 323, and 322 and reduced contributions from typical low‐mass fragments, consistent with the influence of unsaturation on gas phase dissociation pathways [4, 11, 16]. The detection of unsaturated analogues extends patterns previously described for other members of the genus. A representative fragmentation pathway for Compound 23 is shown in Figure 5.

The highest mass compound detected, Compound 11 ([M + H]^+^, *m/z* 601.4679), produced a complex MS/MS spectrum containing both canonical low‐mass ions and higher mass fragments at *m/z* 224 and 165. Late‐eluting compounds such as 23 and 26 exhibited atypical fragmentation profiles with reduced relative intensities of characteristic spermine‐derived ions, suggesting more extensively modified acyl residues. Although definitive structural elucidation would require isolation and NMR analysis, HRMS/MS data establish their close relationship to known budmunchiamines and indicate the presence of previously unreported analogues [24].

Taken together, the UHPLC–HRMS/MS dataset demonstrates that *A. niopoides* is a rich source of macrocyclic spermine alkaloids organized as homologous series defined by systematic variation in fatty acyl substituents. The conserved fragmentation features observed across all compounds provide strong evidence for their assignment to the budmunchiamine class, whereas comparison with literature data confirms the presence of known metabolites alongside numerous new analogues [24]. These findings expand the documented chemical diversity of *A. niopoides* and highlight the effectiveness of high‐resolution tandem mass spectrometry for dereplication and comparative phytochemical analysis of complex polyamine alkaloid mixtures.

## 4 Conclusions

High‐resolution ultrahigh‐performance liquid chromatography coupled with electrospray ionization quadrupole time‐of‐flight mass spectrometry (UHPLC–ESI–QTOF–MS/MS) enabled comprehensive characterization of the macrocyclic spermine alkaloid profile of *Albizia niopoides* without the need for prior isolation. Accurate mass measurements, sub 5‐ppm errors, and highly conserved CID fragmentation pathways allowed confident annotation of 14 budmunchiamine‐type alkaloids directly from the crude extract.

The consistent appearance of diagnostic polyamine derived fragment ions and characteristic neutral losses confirms that tandem mass spectrometry provides a robust, structure informative approach for dereplication of budmunchiamines across *Albizia* species. Variations in acyl chain length, oxidation state, and unsaturation were readily distinguished based on precursor ion masses, retention times, and MS/MS patterns, highlighting the sensitivity of HRMS/MS to subtle structural modifications within this alkaloid class.

These results underscore the value of high‐resolution tandem mass spectrometry as a primary analytical strategy for surveying complex polyamine alkaloid mixtures and for expanding chemotaxonomic and phytochemical knowledge of underexplored *Albizia* species. Analysis of the dataset from this study, and after a literature review of alkaloids isolated from species of the genus *Albizia*, suggests 20 new budmunchiamine‐type alkaloid structures in the *A. niopoides* extract.

## Author Contributions

**Maria L. A. Majevski:** investigation. **Lienne D'Auria Lima:** investigation. **Adriana C. C. Reis:** investigation, methodology, formal analysis, writing – original draft. **Leonan I. C. R. Santos:** investigation, formal analysis, writing – original draft. **Brenno F. S. Vargas:** investigation, formal analysis, writing – original draft. **Markus Kohlhoff:** investigation, writing – review and editing, formal analysis, supervision. **Geraldo Célio Brandão:** investigation, funding acquisition, writing – original draft, writing – review and editing.

## Funding

This study was supported by Fundação de Amparo à Pesquisa do Estado de Minas Gerais (grant no. APQ‐01375–24, BPD‐00381–22, RED‐00110‐23, and RED‐00170–23), FAPEMIG Research Productivity Scholarship (grant no. APQ‐06528–24), National Council for Scientific and Technological Development (CNPq) (grants no. 402010/2023–0).

## Conflicts of Interest

The authors declare no conflicts of interest.

## Supporting information




**Figure S1:** UV spectrum of macrocyclic spermine based present in the ethanolic extract alkaloids of *A. niopoides* trunk.
**Figure S2:** MS^1^ spectrum of macrocyclic spermine based present in the ethanolic extract alkaloids of *A. niopoides* trunk.
**Figure S3:** MS^2^ spectrum of macrocyclic spermine based present in the ethanolic extract alkaloids of *A. niopoides* trunk.
**Figure S4:** Fragmentation pathways of macrocyclic spermine based (Compound 1).
**Figure S5:** UV spectrum of macrocyclic spermine based present in the ethanolic extract alkaloids of *A. niopoides* trunk.
**Figure S6:** MS^1^ spectrum of macrocyclic spermine based present in the ethanolic extract alkaloids of *A. niopoides* trunk.
**Figure S7:** MS^2^ spectrum of macrocyclic spermine based present in the ethanolic extract alkaloids of *A. niopoides* trunk.
**Figure S8:** Fragmentation pathways of macrocyclic spermine based (Compound 2).
**Figure S9:** UV spectrum of macrocyclic spermine based present in the ethanolic extract alkaloids of *A. niopoides* trunk.
**Figure S10:** MS^1^ spectrum of macrocyclic spermine based present in the ethanolic extract alkaloids of *A. niopoides* trunk.
**Figure S11:** MS^2^ spectrum of macrocyclic spermine based present in the ethanolic extract alkaloids of *A. niopoides* trunk.
**Figure S12:** Fragmentation pathways of macrocyclic spermine based (Compound 3).
**Figure S13:** UV spectrum of macrocyclic spermine based present in the ethanolic extract alkaloids of *A. niopoides* trunk.
**Figure S14:** MS^1^ spectrum of macrocyclic spermine based present in the ethanolic extract alkaloids of *A. niopoides* trunk.
**Figure S15:** MS^2^ spectrum of macrocyclic spermine based present in the ethanolic extract alkaloids of *A. niopoides* trunk.
**Figure S16:** UV spectrum of macrocyclic spermine based present in the ethanolic extract alkaloids of *A. niopoides* trunk.
**Figure S17:** MS^1^ spectrum of macrocyclic spermine based present in the ethanolic extract alkaloids of *A. niopoides* trunk.
**Figure S18:** MS^2^ spectrum of macrocyclic spermine based present in the ethanolic extract alkaloids of *A. niopoides* trunk.
**Figure S19:** Fragmentation pathways of macrocyclic spermine based (Compound 5).
**Figure S20:** UV spectrum of macrocyclic spermine based present in the ethanolic extract alkaloids of *A. niopoides* trunk.
**Figure S21:** MS^2^ spectrum of macrocyclic spermine based present in the ethanolic extract alkaloids of *A. niopoides* trunk.
**Figure S22:** MS^1^ spectrum of macrocyclic spermine based present in the ethanolic extract alkaloids of *A. niopoides* trunk.
**Figure S23:** Fragmentation pathways of macrocyclic spermine based (Compound 6).
**Figure S24:** UV spectrum of macrocyclic spermine based present in the ethanolic extract alkaloids of *A. niopoides* trunk.
**Figure S25:** MS^1^ spectrum of macrocyclic spermine based present in the ethanolic extract alkaloids of *A. niopoides* trunk.
**Figure S26:** MS^2^ spectrum of macrocyclic spermine based present in the ethanolic extract alkaloids of *A. niopoides* trunk.
**Figure S27:** Fragmentation pathways of macrocyclic spermine based (Compound 7).
**Figure S28:** UV spectrum of macrocyclic spermine based present in the ethanolic extract alkaloids of *A. niopoides* trunk.
**Figure S29:** MS^1^ spectrum of macrocyclic spermine based present in the ethanolic extract alkaloids of *A. niopoides* trunk.
**Figure S30:** MS^2^ spectrum of macrocyclic spermine based present in the ethanolic extract alkaloids of *A. niopoides* trunk.
**Figure S31:** Fragmentation pathways of macrocyclic spermine based (Compound 6).
**Figure S32:** UV spectrum of macrocyclic spermine based present in the ethanolic extract alkaloids of *A. niopoides* trunk.
**Figure S33:** MS^1^ spectrum of macrocyclic spermine based present in the ethanolic extract alkaloids of *A. niopoides* trunk.
**Figure S34:** MS^2^ spectrum of macrocyclic spermine based present in the ethanolic extract alkaloids of *A. niopoides* trunk.
**Figure S35:** Fragmentation pathways of macrocyclic spermine based (Compound 9).
**Figure S36:** UV spectrum of macrocyclic spermine based present in the ethanolic extract alkaloids of *A. niopoides* trunk.
**Figure S37:** MS^1^ spectrum of macrocyclic spermine based present in the ethanolic extract alkaloids of *A. niopoides* trunk.
**Figure S38:** MS^2^ spectrum of macrocyclic spermine based present in the ethanolic extract alkaloids of *A. niopoides* trunk.
**Figure S39:** Fragmentation pathways of macrocyclic spermine based (Compound 10).
**Figure S40:** UV spectrum of macrocyclic spermine based present in the ethanolic extract alkaloids of *A. niopoides* trunk.
**Figure S41:** MS^1^ spectrum of macrocyclic spermine based present in the ethanolic extract alkaloids of *A. niopoides* trunk.
**Figure S42:** MS^2^ spectrum of macrocyclic spermine based present in the ethanolic extract alkaloids of *A. niopoides* trunk.
**Figure S43:** Fragmentation pathways of macrocyclic spermine based (Compound 11).
**Figure S44:** UV spectrum of macrocyclic spermine based present in the ethanolic extract alkaloids of *A. niopoides* trunk.
**Figure S45:** MS^1^ spectrum of macrocyclic spermine based present in the ethanolic extract alkaloids of *A. niopoides* trunk.
**Figure S46:** MS^2^ spectrum of macrocyclic spermine based present in the ethanolic extract alkaloids of *A. niopoides* trunk.
**Figure S47:** UV spectrum of macrocyclic spermine based present in the ethanolic extract alkaloids of *A. niopoides* trunk.
**Figure S48:** MS^1^ spectrum of macrocyclic spermine based present in the ethanolic extract alkaloids of *A. niopoides* trunk.
**Figure S49:** MS^2^ spectrum of macrocyclic spermine based present in the ethanolic extract alkaloids of *A. niopoides* trunk.
**Figure S50:** Fragmentation pathways of macrocyclic spermine based (Compound 14).
**Figure S51:** UV spectrum of macrocyclic spermine based present in the ethanolic extract alkaloids of *A. niopoides* trunk.
**Figure S52:** MS^1^ spectrum of macrocyclic spermine based present in the ethanolic extract alkaloids of *A. niopoides* trunk.
**Figure S53:** MS^2^ spectrum of macrocyclic spermine based present in the ethanolic extract alkaloids of *A. niopoides* trunk.
**Figure S54:** Fragmentation pathways of macrocyclic spermine based (Compound 14).
**Figure S55:** UV spectrum of macrocyclic spermine based present in the ethanolic extract alkaloids of *A. niopoides* trunk.
**Figure S56:** MS^1^ spectrum of macrocyclic spermine based present in the ethanolic extract alkaloids of *A. niopoides* trunk.
**Figure S57:** MS^2^ spectrum of macrocyclic spermine based present in the ethanolic extract alkaloids of *A. niopoides* trunk.
**Figure S58:** Fragmentation pathways of macrocyclic spermine based (Compound 15).
**Figure S59:** UV spectrum of macrocyclic spermine based present in the ethanolic extract alkaloids of *A. niopoides* trunk.
**Figure S60:** MS^1^ spectrum of macrocyclic spermine based present in the ethanolic extract alkaloids of *A. niopoides* trunk.
**Figure S61:** MS^2^ spectrum of macrocyclic spermine based present in the ethanolic extract alkaloids of *A. niopoides* trunk.
**Figure S62:** Fragmentation pathways of macrocyclic spermine based (Compound 16).
**Figure S63:** UV spectrum of macrocyclic spermine based present in the ethanolic extract alkaloids of *A. niopoides* trunk.
**Figure S64:** MS^1^ spectrum of macrocyclic spermine based present in the ethanolic extract alkaloids of *A. niopoides* trunk.
**Figure S65:** MS^2^ spectrum of macrocyclic spermine based present in the ethanolic extract alkaloids of *A. niopoides* trunk.
**Figure S66:** Fragmentation pathways of macrocyclic spermine based (Compound 17).
**Figure S67:** UV spectrum of macrocyclic spermine based present in the ethanolic extract alkaloids of *A. niopoides* trunk.
**Figure S68:** MS^1^ spectrum of macrocyclic spermine based present in the ethanolic extract alkaloids of *A. niopoides* trunk.
**Figure S69:** MS^2^ spectrum of macrocyclic spermine based present in the ethanolic extract alkaloids of *A. niopoides* trunk.
**Figure S70:** Fragmentation pathways of macrocyclic spermine based (Compound 18).
**Figure S71:** UV spectrum of macrocyclic spermine based present in the ethanolic extract alkaloids of *A. niopoides* trunk.
**Figure S72:** MS^1^ spectrum of macrocyclic spermine based present in the ethanolic extract alkaloids of *A. niopoides* trunk.
**Figure S73:** MS^2^ spectrum of macrocyclic spermine based present in the ethanolic extract alkaloids of *A. niopoides* trunk.
**Figure S74:** Fragmentation pathways of macrocyclic spermine based (Compound 19).
**Figure S75:** UV spectrum of macrocyclic spermine based present in the ethanolic extract alkaloids of *A. niopoides* trunk.
**Figure S76:** MS^1^ spectrum of macrocyclic spermine based present in the ethanolic extract alkaloids of *A. niopoides* trunk.
**Figure S77:** MS^2^ spectrum of macrocyclic spermine based present in the ethanolic extract alkaloids of *A. niopoides* trunk.
**Figure S78:** Fragmentation pathways of macrocyclic spermine based (Compound 20).
**Figure S79:** UV spectrum of macrocyclic spermine based present in the ethanolic extract alkaloids of *A. niopoides* trunk.
**Figure S80:** MS^1^ spectrum of macrocyclic spermine based present in the ethanolic extract alkaloids of *A. niopoides* trunk.
**Figure S81:** MS^2^ spectrum of macrocyclic spermine based present in the ethanolic extract alkaloids of *A. niopoides* trunk.
**Figure S82:** Fragmentation pathways of macrocyclic spermine based (Compound 21).
**Figure S83:** UV spectrum of macrocyclic spermine based present in the ethanolic extract alkaloids of *A. niopoides* trunk.
**Figure S84:** MS^1^ spectrum of macrocyclic spermine based present in the ethanolic extract alkaloids of *A. niopoides* trunk.
**Figure S85:** MS^2^ spectrum of macrocyclic spermine based present in the ethanolic extract alkaloids of *A. niopoides* trunk.
**Figure S86:** Fragmentation pathways of macrocyclic spermine based (Compound 22).
**Figure S87:** UV spectrum of macrocyclic spermine based present in the ethanolic extract alkaloids of *A. niopoides* trunk.
**Figure S88:** MS^1^ spectrum of macrocyclic spermine based present in the ethanolic extract alkaloids of *A. niopoides* trunk.
**Figure S89:** MS^2^ spectrum of macrocyclic spermine based present in the ethanolic extract alkaloids of *A. niopoides* trunk.
**Figure S90:** UV spectrum of macrocyclic spermine based present in the ethanolic extract alkaloids of *A. niopoides* trunk.
**Figure S91:** MS^1^ spectrum of macrocyclic spermine based present in the ethanolic extract alkaloids of *A. niopoides* trunk.
**Figure S92:** MS^2^ spectrum of macrocyclic spermine based present in the ethanolic extract alkaloids of *A. niopoides* trunk.
**Figure S93:** Fragmentation pathways of macrocyclic spermine based (Compound 24).
**Figure S94:** UV spectrum of macrocyclic spermine based present in the ethanolic extract alkaloids of *A. niopoides* trunk.
**Figure S95:** MS^1^ spectrum of macrocyclic spermine based present in the ethanolic extract alkaloids of *A. niopoides* trunk.
**Figure S96:** MS^2^ spectrum of macrocyclic spermine based present in the ethanolic extract alkaloids of *A. niopoides* trunk.
**Figure S97:** Fragmentation pathways of macrocyclic spermine based (Compound 25).
**Figure S98:** UV spectrum of macrocyclic spermine based present in the ethanolic extract alkaloids of *A. niopoides* trunk.
**Figure S99:** MS^1^ spectrum of macrocyclic spermine based present in the ethanolic extract alkaloids of *A. niopoides* trunk.
**Figure S100:** MS^2^ spectrum of macrocyclic spermine based present in the ethanolic extract alkaloids of *A. niopoides* trunk.
**Figure S101:** Fragmentation pathways of macrocyclic spermine based (Compound 26).

## Data Availability

The data that supports the findings of this study are available in the Supporting Information of this article.
