# Supplementary material for: High‐Resolution LC–MS/MS Characterization of Budmunchiamine‐Type Alkaloids in Albizia niopoides
Source: Rapid Commun Mass Spectrom. 2026 Jul 25;40(19):e70142. doi: 10.1002/rcm.70142 (PMC13401227; doi:10.1002/rcm.70142)
Supplement: Supplementary file 1 — Figure S1: UV spectrum of macrocyclic spermine based present in the ethanolic extract alkaloids of A. niopoides trunk. Figure S2: MS1 spectrum of macrocyclic spermine based present in the ethanolic extract alkaloids of A. niopoides trunk. Figure S3: MS2 spectrum of macrocyclic spermine based present in the ethanolic extract alkaloids of A. niopoides trunk. Figure S4: Fragmentation pathways of macrocyclic spermine based (Compound 1). Figure S5: UV spectrum of macrocyclic spermine based present in the ethanolic extract alkaloids of A. niopoides trunk. Figure S6: MS1 spectrum of macrocyclic spermine based present in the ethanolic extract alkaloids of A. niopoides trunk. Figure S7: MS2 spectrum of macrocyclic spermine based present in the ethanolic extract alkaloids of A. niopoides trunk. Figure S8: Fragmentation pathways of macrocyclic spermine based (Compound 2). Figure S9: UV spectrum of macrocyclic spermine based present in the ethanolic extract alkaloids of A. niopoides trunk. Figure S10: MS1 spectrum of macrocyclic spermine based present in the ethanolic extract alkaloids of A. niopoides trunk. Figure S11: MS2 spectrum of macrocyclic spermine based present in the ethanolic extract alkaloids of A. niopoides trunk. Figure S12: Fragmentation pathways of macrocyclic spermine based (Compound 3). Figure S13: UV spectrum of macrocyclic spermine based present in the ethanolic extract alkaloids of A. niopoides trunk. Figure S14: MS1 spectrum of macrocyclic spermine based present in the ethanolic extract alkaloids of A. niopoides trunk. Figure S15: MS2 spectrum of macrocyclic spermine based present in the ethanolic extract alkaloids of A. niopoides trunk. Figure S16: UV spectrum of macrocyclic spermine based present in the ethanolic extract alkaloids of A. niopoides trunk. Figure S17: MS1 spectrum of macrocyclic spermine based present in the ethanolic extract alkaloids of A. niopoides trunk. Figure S18: MS2 spectrum of macrocyclic spermine based present in the ethanolic ext [file RCM-40-e70142-s001.docx]

**High Resolution LC–MS/MS Characterization of Budmunchiamine Type Alkaloids in *Albizia niopoides***

Maria L. A. Majevski¹, Lienne D'Auria Lima¹, Adriana C. C. Reis¹, Leonan I. C. R. Santos¹, Brenno F. S. Vargas¹, Markus Kohlhoff², and Geraldo Célio Brandão¹,*

¹Escola de Farmácia, Universidade Federal de Ouro Preto, Campus Morro do Cruzeiro, Ouro Preto 35.402-163, Minas Gerais, Brazil

²Laboratório de Química de Produtos Naturais Bioativos, Fundação Oswaldo Cruz, Instituto René Rachou, Belo Horizonte 30.190-009, Minas Gerais, Brazil

*Correspondence: [celiobrandao@ufop.edu.br](mailto:celiobrandao@ufop.edu.br); Tel.: +55-31-3551-1088; Fax: +55-31-3551-1069

**Compound (1) RT 1.1 min. MM 427 Da**

In the first fragmentation step, the protonated molecular ion (m/z 427) undergoes an inductive cleavage at the amine moiety concomitantly with an α-cleavage adjacent to the lactone ring, resulting in the elimination of a neutral fragment assigned as C₃H₁₀N₂ (74 Da). The resulting product ion subsequently undergoes an additional charge-induced cleavage, yielding the fragment observed at m/z 282. Further dissociation is proposed to involve a rearrangement process accompanied by the formation of a neutral furan species, generating the ion at m/z 214 (Figure 4s).

**
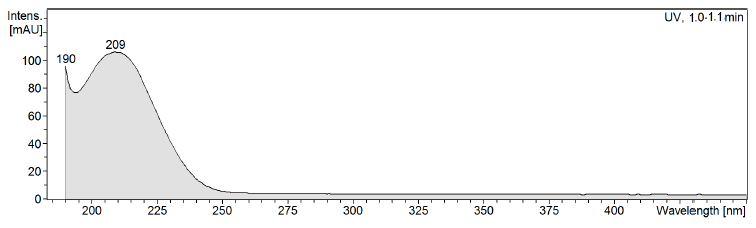
**

Figure 1S. UV spectrum of macrocyclic spermine based present in the ethanolic extract alkaloids of *A. niopoides* trunk


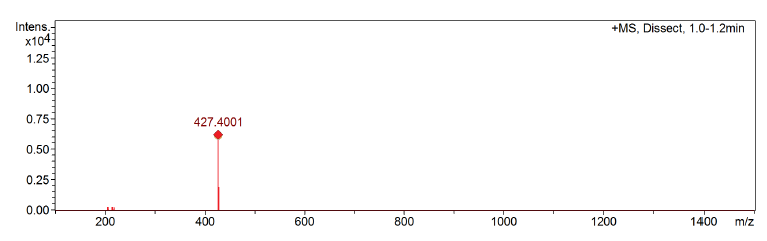


Figure 2S. MS^1^ spectrum of macrocyclic spermine based present in the ethanolic extract alkaloids of *A. niopoides* trunk


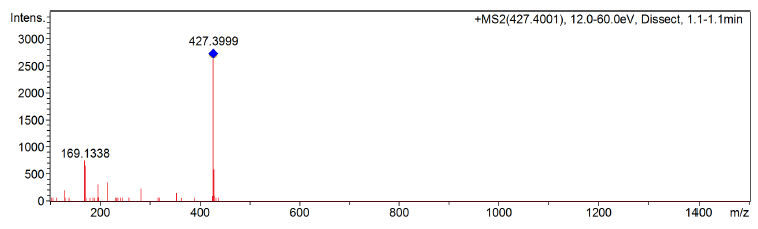

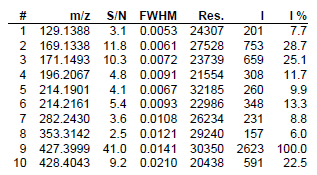


Figure 3S. MS^2^ spectrum of macrocyclic spermine based present in the ethanolic extract alkaloids of *A. niopoides* trunk

Figure 4S. Fragmentation pathways of macrocyclic spermine based (Compound 1).

**Compound (2) RT 3.1 min. MM 427 Da**


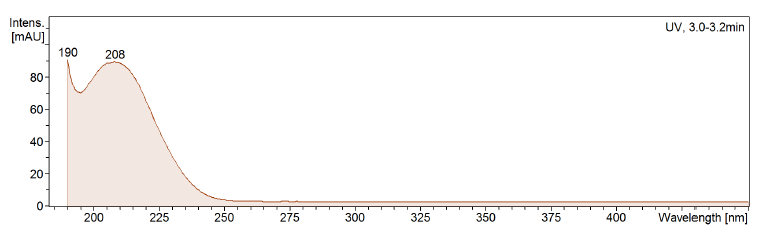


Figure 5S. UV spectrum of macrocyclic spermine based present in the ethanolic extract alkaloids of *A. niopoides* trunk

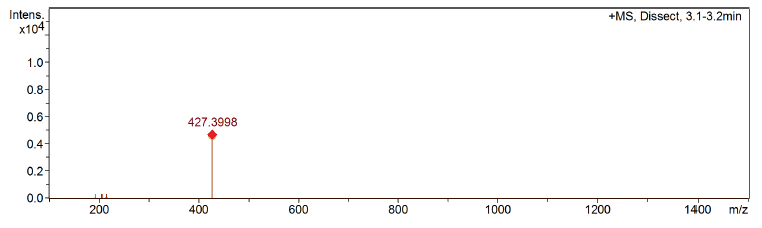


Figure 6S. MS^1^ spectrum of macrocyclic spermine based present in the ethanolic extract alkaloids of *A. niopoides* trunk


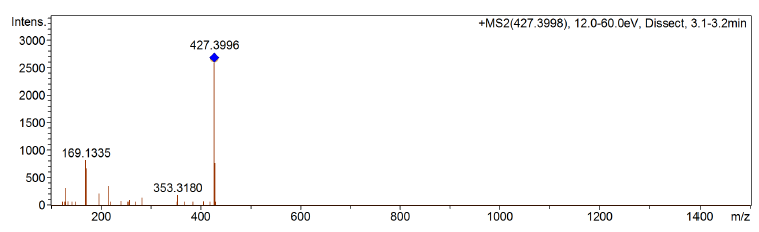


Figure 7S. MS^2^ spectrum of macrocyclic spermine based present in the ethanolic extract alkaloids of *A. niopoides* trunk


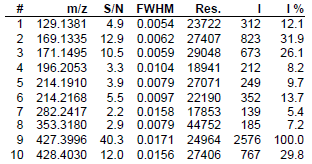

Figure 8S. Fragmentation pathways of macrocyclic spermine based (Compound 2).

**Compound (3) RT 3.7 min. MM 499 Da**

In the first fragmentation step, the protonated molecular ion (m/z 499) undergoes an inductive cleavage at the amine moiety concomitantly with an α-cleavage adjacent to the lactone ring, resulting in the elimination of a neutral fragment assigned as C₃H₁₀N₂ (74 Da). The resulting product ion subsequently undergoes an additional charge-induced cleavage **simultaneously with** neutral furan formation and dehydration **in the** lateral chain, yielding the fragment observed at m/z 268. Subsequently, the ion undergoes dehydration producing the fragment ion at m/z 250, forming a conjugated alkene (Figure 11S and 12 S).


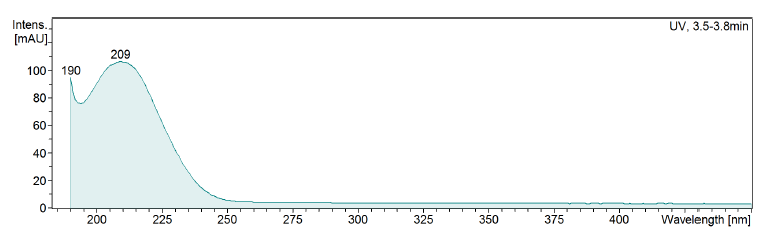


Figure 9S. UV spectrum of macrocyclic spermine based present in the ethanolic extract alkaloids of *A. niopoides* trunk


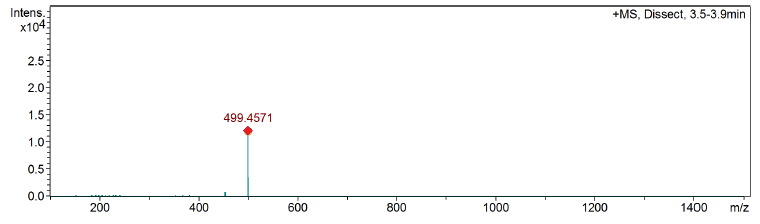


Figure 10S. MS^1^ spectrum of macrocyclic spermine based present in the ethanolic extract alkaloids of *A. niopoides* trunk


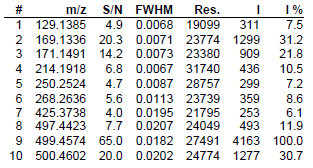

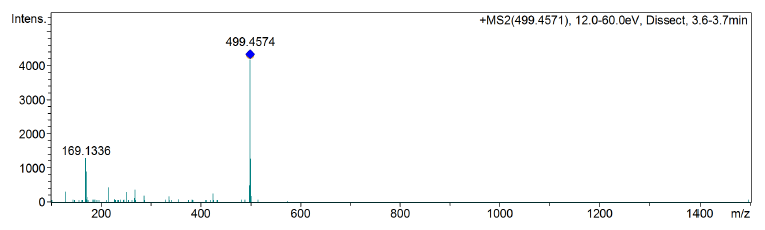


Figure 11S. MS^2^ spectrum of macrocyclic spermine based present in the ethanolic extract alkaloids of *A. niopoides* trunk

Figure 12S. Fragmentation pathways of macrocyclic spermine based (Compound 3).

**Compound (4) RT 3.8 min. MM 455 Da**

The fragmentation process is initiated by dehydration of the precursor ion at m/z 455, affording the product ion at m/z 437. Subsequent loss of ammonia yields the fragment ion at m/z 420. In a competing fragmentation pathway, cleavage of the side chain adjacent to the double bond results in the neutral loss of a C₃H₆ moiety, producing the ion at m/z 395. This fragmentation pathway provides valuable information regarding the position of the hydroxyl group in the side chain. Further sequential fragmentation reactions, including the loss of CH₂ units, side-chain fragments, and portions of the macrocyclic ring, ultimately generate the characteristic ion at m/z 169 (Figure 4 and 15S).


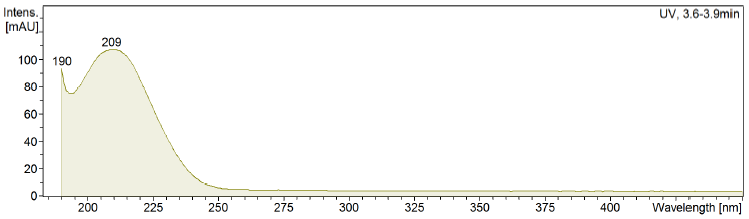


Figure 13S. UV spectrum of macrocyclic spermine based present in the ethanolic extract alkaloids of *A. niopoides* trunk


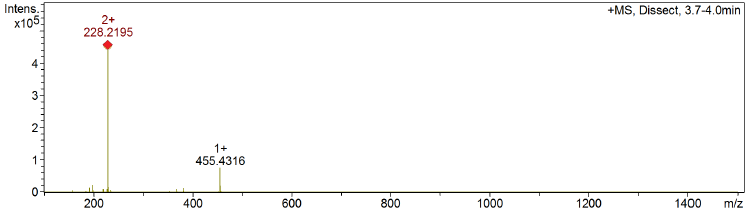


Figure 14S. MS^1^ spectrum of macrocyclic spermine based present in the ethanolic extract alkaloids of *A. niopoides* trunk


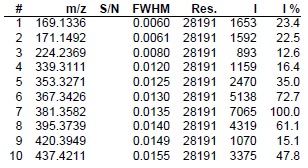

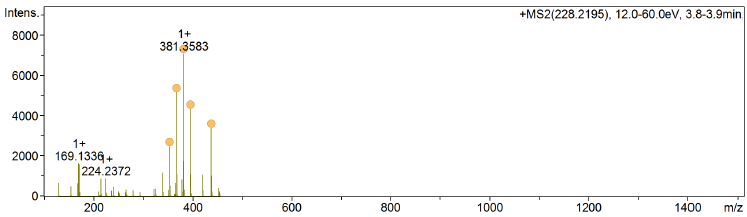


Figure 15S. MS^2^ spectrum of macrocyclic spermine based present in the ethanolic extract alkaloids of *A. niopoides* trunk

**Compound (5) RT 4.1 min. MM 527 Da**

The fragmentation pathway is initiated by cleavage of the protonated molecular ion (m/z 527), involving opening of the macrocyclic ring followed by dehydration, ultimately yielding the product ion at m/z 296. An alternative dissociation pathway involves cleavage of the saturated side chain in combination with the characteristic bond cleavages within the macrocyclic framework, resulting in the neutral loss of a fragment with the molecular formula C₁₉H₃₉NO₂. The sequential fragmentation events observed thereafter are consistent with the well-established dissociation behavior of this class of macrocyclic polyamines and have been extensively reported in this study (Figure 18S and 19S).


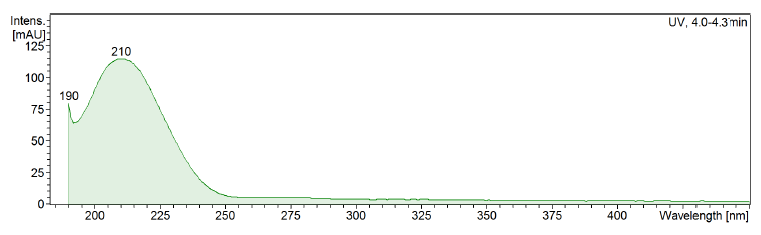


Figure 16S. UV spectrum of macrocyclic spermine based present in the ethanolic extract alkaloids of *A. niopoides* trunk


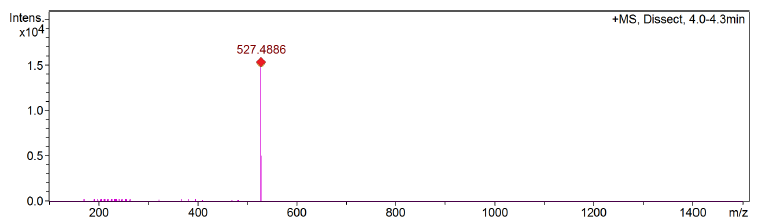


Figure 17S. MS^1^ spectrum of macrocyclic spermine based present in the ethanolic extract alkaloids of *A. niopoides* trunk


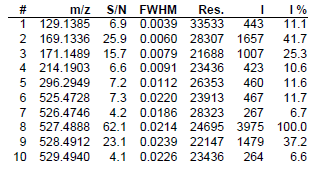


Figure 18S. MS^2^ spectrum of macrocyclic spermine based present in the ethanolic extract alkaloids of *A. niopoides* trunk


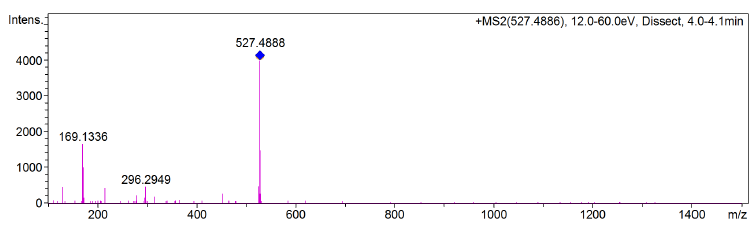

Figure 19S. Fragmentation pathways of macrocyclic spermine based (Compound 5).

**Compound (6) RT 4.2 min. MM 411 Da**

One of the proposed fragmentation pathways involves the elimination of the amide nitrogen as ammonia, resulting in a neutral loss of 17 Da. An alternative and highly conserved dissociation route for this class of compounds proceeds through a combination of charge-induced and α-cleavages at the amide and macrocyclic nitrogen atoms, leading to the elimination of a 74 Da side chain. Subsequent α-cleavage followed by the neutral loss of carbon monoxide (CO) generates the product ion at m/z 238. Finally, sequential eliminations of neutral methylene (CH₂) and acetylene (C₂H₂) units give rise to the fragment ion observed at m/z 184, completing one of the characteristic fragmentation pathways of this family of macrocyclic compounds (Figure 22S and 23S).

**
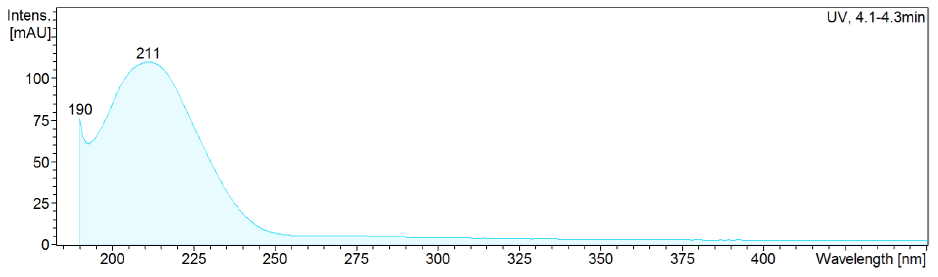
**

Figure 20S. UV spectrum of macrocyclic spermine based present in the ethanolic extract alkaloids of *A. niopoides* trunk

**
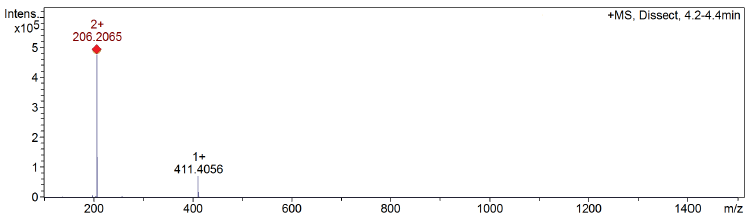
**

Figure 21S. MS^2^ spectrum of macrocyclic spermine based present in the ethanolic extract alkaloids of *A. niopoides* trunk

**
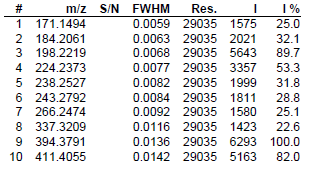
**

Figure 22S. MS^1^ spectrum of macrocyclic spermine based present in the ethanolic extract alkaloids of *A. niopoides* trunk

**
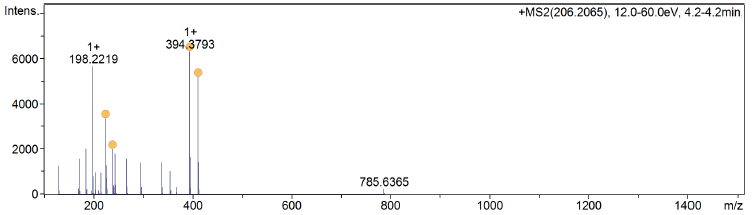
**

Figure 23S. Fragmentation pathways of macrocyclic spermine based (Compound 6).

**Compound (7) RT 4.3 min. MM 483 Da**

The fragmentation pathway is initiated by dehydration of the terminal alcohol, resulting in the neutral loss of 18 Da and the formation of an unsaturated intermediate. Subsequent cleavage of the neutral propylene bridge connecting the nitrogen atoms generates the product ion at *m/z* 423. Further sequential losses of CH₂ units from the side chain give rise to the fragment ion at *m/z* 339. These stepwise eliminations are consistent with the position of the terminal alkene formed during the initial dehydration and therefore provide additional evidence supporting the location of the original hydroxyl group.

The ion at *m/z* 339 subsequently undergoes fragmentation through cleavage of the aliphatic side chain in combination with characteristic bond cleavages within the macrocyclic framework, ultimately yielding the diagnostic fragment ion at *m/z* 171. The observed fragmentation pattern is consistent with the established dissociation behavior of macrocyclic polyamines and provides structural information for the assignment of the side-chain connectivity (Figure 26S and 27S).


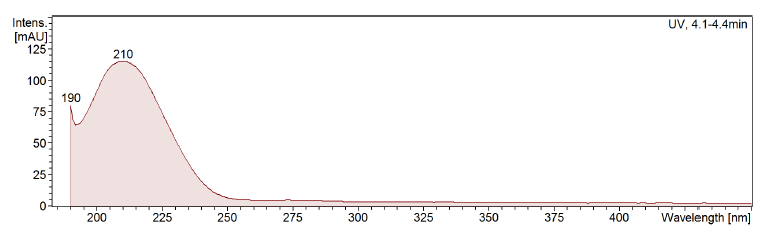


Figure 24S. UV spectrum of macrocyclic spermine based present in the ethanolic extract alkaloids of *A. niopoides* trunk


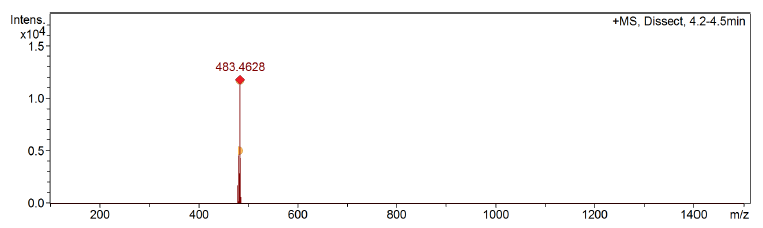


Figure 25S. MS^1^ spectrum of macrocyclic spermine based present in the ethanolic extract alkaloids of *A. niopoides* trunk


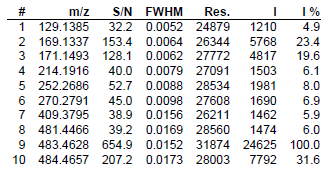

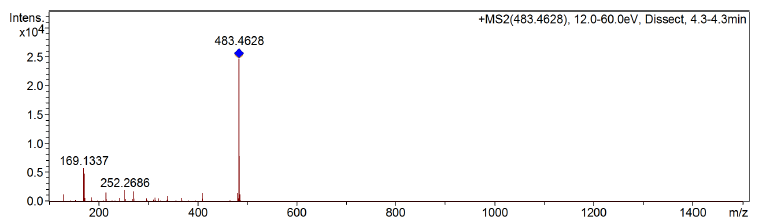


Figure 26S. MS^2^ spectrum of macrocyclic spermine based present in the ethanolic extract alkaloids of *A. niopoides* trunk

Figure 27S. Fragmentation pathways of macrocyclic spermine based (Compound 7).

**Compound (8) RT 4.5 min. MM 555 Da**

The compound undergoes two distinct fragmentation pathways. One of these represents a highly conserved dissociation mechanism observed throughout this class of macrocyclic polyamines, involving the elimination of the side chain followed by concurrent α-cleavage and charge-induced cleavage between the macrocyclic nitrogen and the amide group, ultimately generating the diagnostic product ion at *m/z* 214. The subsequent fragmentation of this ion follows the same dissociation sequence discussed previously.

An alternative fragmentation pathway involves cleavage of the macrocyclic ring accompanied by dehydration of the side chain, resulting in the neutral loss of a fragment with the molecular formula C₁₁H₂₅N₃O₂. A subsequent dehydration step produces the product ion at *m/z* 306, which is tentatively assigned to a structure containing a conjugated diene system (Figure 30S and 31S).


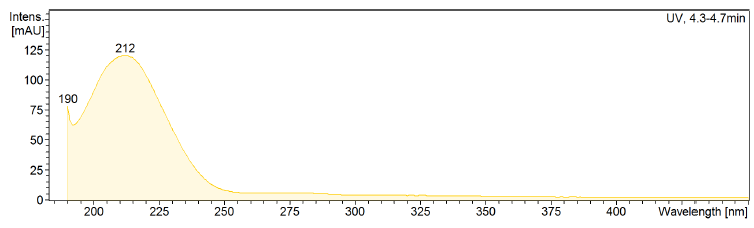


Figure 28S. UV spectrum of macrocyclic spermine based present in the ethanolic extract alkaloids of *A. niopoides* trunk


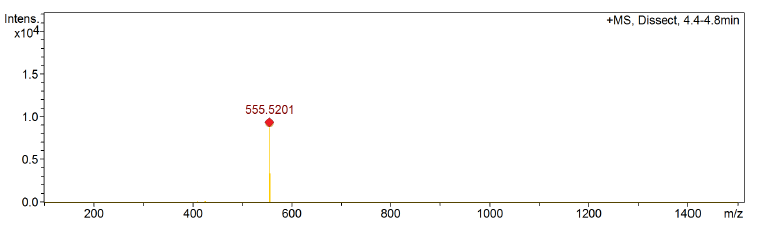


Figure 29S. MS^1^ spectrum of macrocyclic spermine based present in the ethanolic extract alkaloids of *A. niopoides* trunk


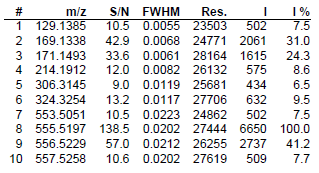

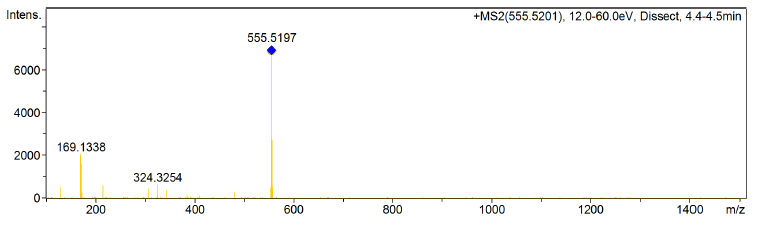


Figure 30S. MS^2^ spectrum of macrocyclic spermine based present in the ethanolic extract alkaloids of *A. niopoides* trunk

Figure 31S. Fragmentation pathways of macrocyclic spermine based (Compound 6).

**Compound (9) RT 4.5 min. MM 425 Da**

The fragmentation pattern follows the conserved dissociation pathway characteristic of this class of macrocyclic compounds. The process is initiated by cleavage of the side chain concomitant with the neutral loss of a C₂H₆N fragment, generating the diagnostic product ion at m/z 214. The subsequent fragmentation steps proceed through the same sequence of dissociation events previously described, ultimately yielding the characteristic low-mass product ions observed for this family of compounds (Figure 34S and 35S).


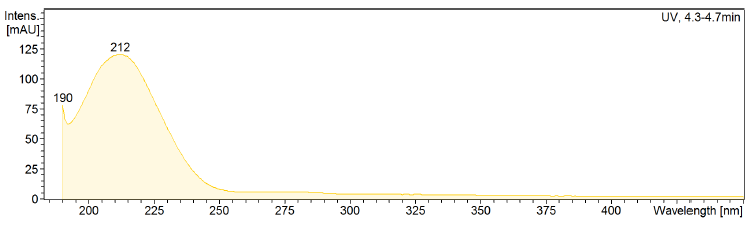


Figure 32S. UV spectrum of macrocyclic spermine based present in the ethanolic extract alkaloids of *A. niopoides* trunk


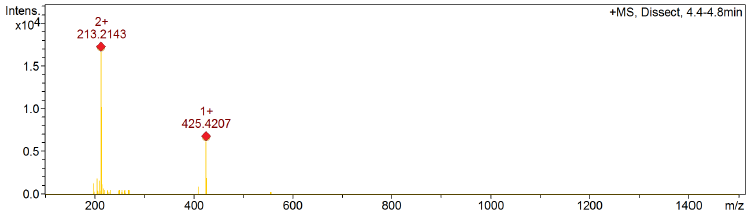


Figure 33S. MS^1^ spectrum of macrocyclic spermine based present in the ethanolic extract alkaloids of *A. niopoides* trunk


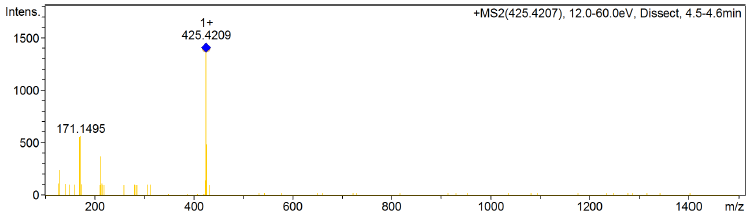


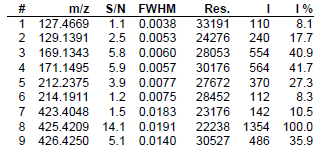


Figure 34S. MS^2^ spectrum of macrocyclic spermine based present in the ethanolic extract alkaloids of *A. niopoides* trunk

Figure 35S. Fragmentation pathways of macrocyclic spermine based (Compound 9).

**Compound (10) RT 4.7 min. MM 439 Da**


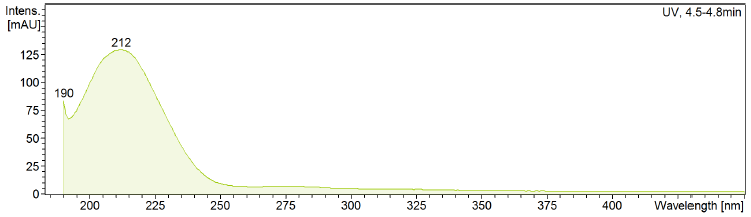


Figure 36S. UV spectrum of macrocyclic spermine based present in the ethanolic extract alkaloids of *A. niopoides* trunk


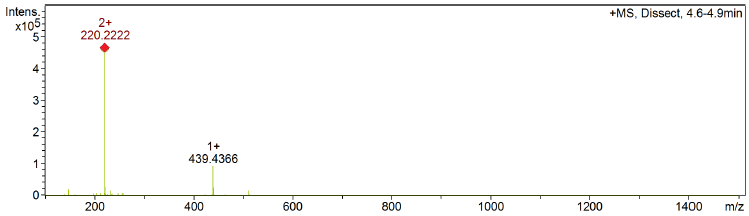


Figure 37S. MS^1^ spectrum of macrocyclic spermine based present in the ethanolic extract alkaloids of *A. niopoides* trunk


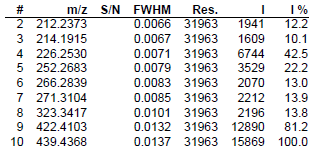

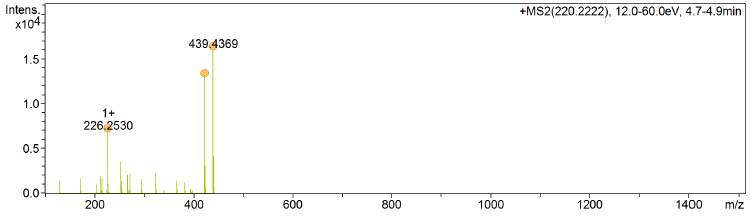


Figure 38S. MS^2^ spectrum of macrocyclic spermine based present in the ethanolic extract alkaloids of *A. niopoides* trunk

Figure 39S. Fragmentation pathways of macrocyclic spermine based (Compound 10).

**Compound (11) RT 4.9 min. MM 601 Da**

The molecular structure undergoes the conserved fragmentation pathway characteristic of this class of compounds, involving cleavage of the side chain together with the neutral loss of a C₂H₆N fragment from the macrocyclic framework, thereby generating the diagnostic product ion at *m/z* 214. The subsequent dissociation events follow the fragmentation sequence previously described for related compounds.

An alternative fragmentation pathway involves cleavage adjacent to one of the double bonds within the side chain, producing the diagnostic ion at *m/z* 165 and allowing the localization of the site of unsaturation. This product ion subsequently undergoes dehydration, corresponding to the neutral loss of 18 Da. No further dehydration of the remaining hydroxyl group is observed, which can be attributed to the formation of a stabilized conjugated π-system together with the absence of a suitable adjacent sp³ hydrogen required to promote an additional elimination reaction (Figure 42S and 43S).


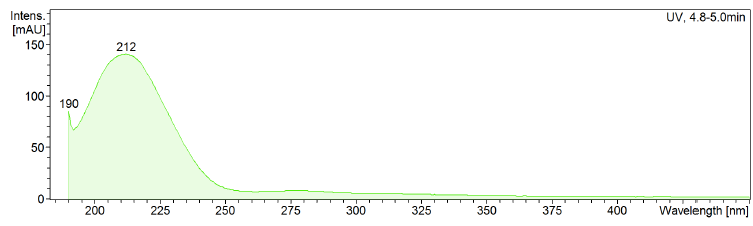


Figure 40S. UV spectrum of macrocyclic spermine based present in the ethanolic extract alkaloids of *A. niopoides* trunk


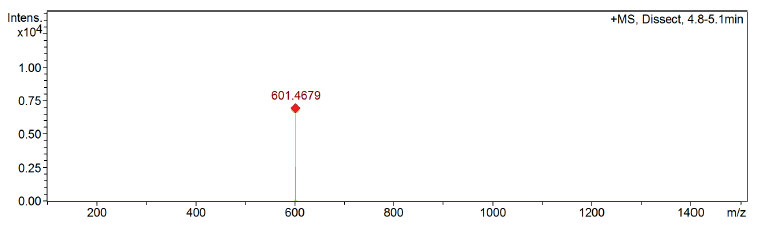


Figure 41S. MS^1^ spectrum of macrocyclic spermine based present in the ethanolic extract alkaloids of *A. niopoides* trunk


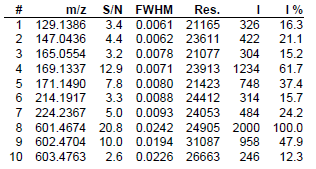

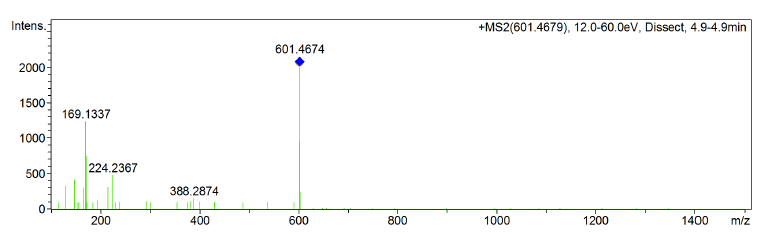


Figure 42S. MS^2^ spectrum of macrocyclic spermine based present in the ethanolic extract alkaloids of *A. niopoides* trunk

Figure 43S. Fragmentation pathways of macrocyclic spermine based (Compound 11).

**Compound (12) RT 5.0 min. MM 465 Da**
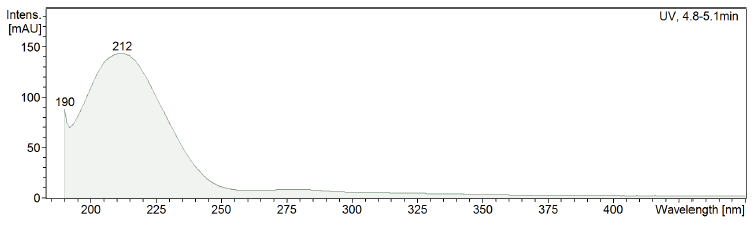


Figure 44S. UV spectrum of macrocyclic spermine based present in the ethanolic extract alkaloids of *A. niopoides* trunk

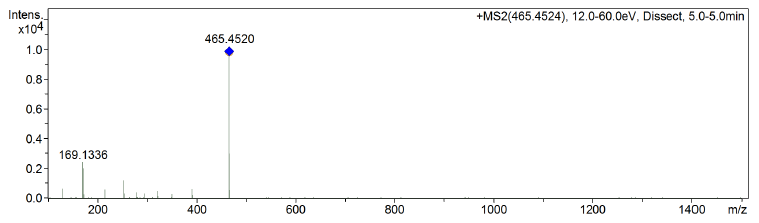

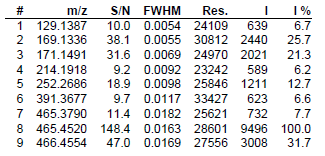

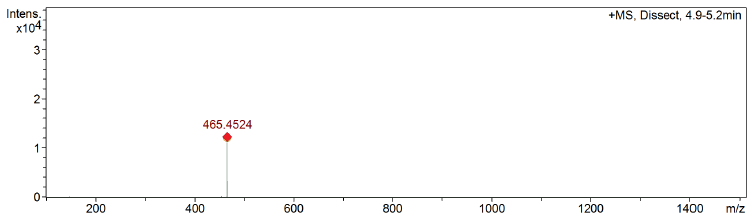


Figure 46S. MS^2^ spectrum of macrocyclic spermine based present in the ethanolic extract alkaloids of *A. niopoides* trunk

Figure 45S. MS^1^ spectrum of macrocyclic spermine based present in the ethanolic extract alkaloids of *A. niopoides* trunk

**Compound (13) RT 5.1 min. MM 539 Da**

The fragmentation pathway is initiated by dehydration of the side-chain hydroxyl group, resulting in the formation of the product ion at *m/z* 521. The terminal alkene generated in this initial step subsequently undergoes cleavage to produce the ion at *m/z* 465, providing diagnostic evidence for the original position of the hydroxyl group through localization of the newly formed double bond. Sequential eliminations of CH₂ units from the unsaturated side chain then yield the product ion at *m/z* 367.

The ion at *m/z* 367 subsequently undergoes the conserved fragmentation pathway characteristic of this class of macrocyclic compounds, involving cleavage of both the side chain and the macrocyclic framework to generate the diagnostic product ion at *m/z* 169. Overall, the observed dissociation pattern is consistent with the established fragmentation behavior and supports the proposed structural assignment of the side chain (Figure 49S and 50S).


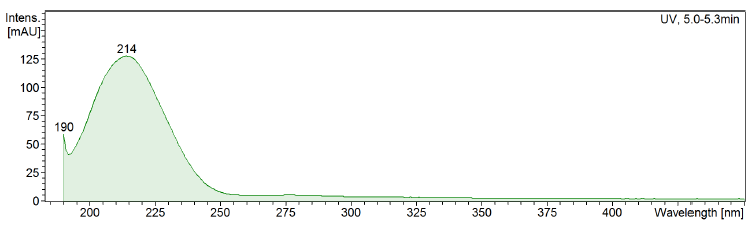


Figure 47S. UV spectrum of macrocyclic spermine based present in the ethanolic extract alkaloids of *A. niopoides* trunk


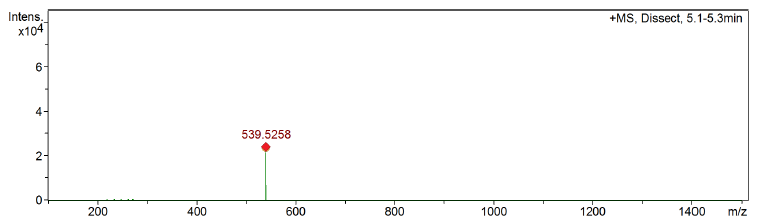


Figure 48S. MS^1^ spectrum of macrocyclic spermine based present in the ethanolic extract alkaloids of *A. niopoides* trunk


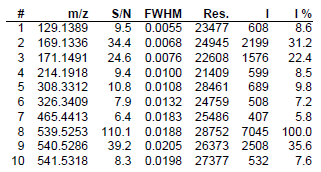

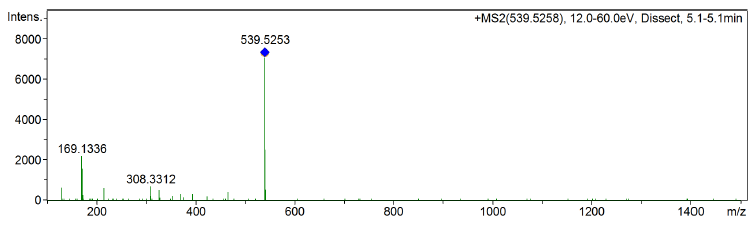


Figure 49S. MS^2^ spectrum of macrocyclic spermine based present in the ethanolic extract alkaloids of *A. niopoides* trunk

Figure 50S. Fragmentation pathways of macrocyclic spermine based (Compound 14).

**Compound (14) RT 5.2 min. MM 457 Da**


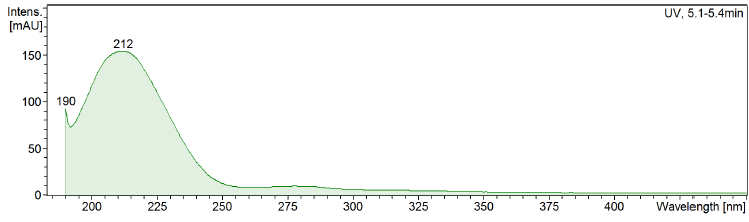


Figure 51S. UV spectrum of macrocyclic spermine based present in the ethanolic extract alkaloids of *A. niopoides* trunk


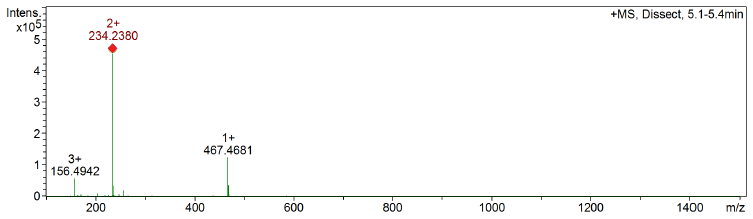


Figure 52S. MS^1^ spectrum of macrocyclic spermine based present in the ethanolic extract alkaloids of *A. niopoides* trunk


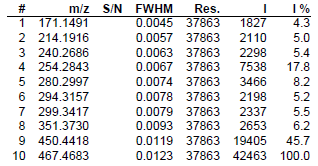

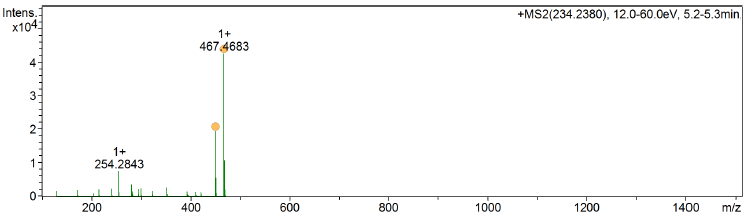


Figure 53S. MS^2^ spectrum of macrocyclic spermine based present in the ethanolic extract alkaloids of *A. niopoides* trunk

Figure 54S. Fragmentation pathways of macrocyclic spermine based (Compound 14).

**Compound (15) RT 5.3 min. MM 421 Da**

The compound exhibits the conserved fragmentation pathways consistently observed throughout the present study. Among the product ions, the fragment at m/z 209 is particularly noteworthy, as it results from cleavage of the macrocyclic framework linked to the biosynthetically derived fatty acid moiety, accompanied by the neutral loss of a CH₂ unit. This dissociation pathway provides diagnostic evidence for the presence of a terminal double bond, thereby supporting the proposed structure of the aliphatic side chain. The remaining fragmentation events are consistent with the characteristic dissociation behavior previously described for this class of macrocyclic polyamines (Figure 57S and 58S).


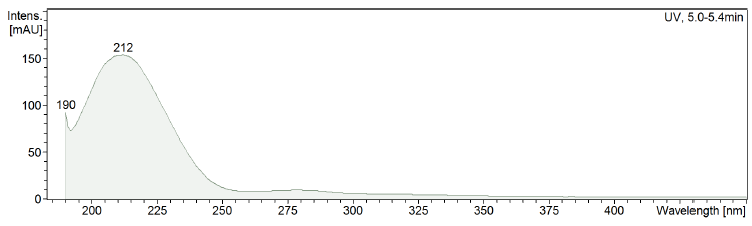


Figure 55S. UV spectrum of macrocyclic spermine based present in the ethanolic extract alkaloids of *A. niopoides* trunk


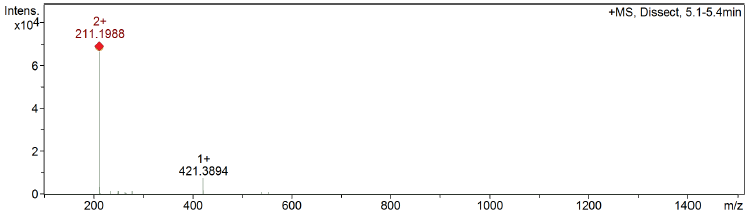


Figure 56S. MS^1^ spectrum of macrocyclic spermine based present in the ethanolic extract alkaloids of *A. niopoides* trunk


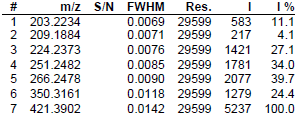

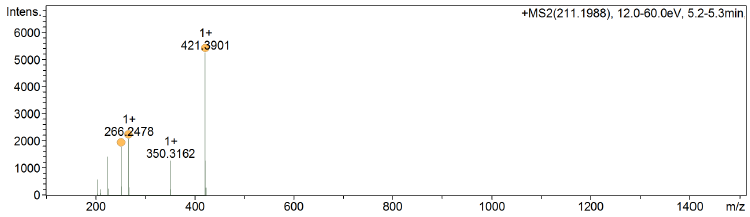


Figure 57S. MS^2^ spectrum of macrocyclic spermine based present in the ethanolic extract alkaloids of *A. niopoides* trunk

Figure 58S. Fragmentation pathways of macrocyclic spermine based (Compound 15).

**Compound (16) RT 5.5 min. MM 481 Da**


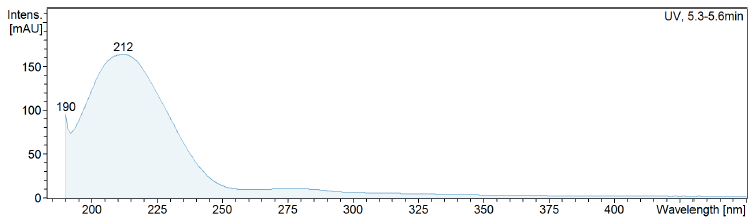


Figure 59S. UV spectrum of macrocyclic spermine based present in the ethanolic extract alkaloids of *A. niopoides* trunk


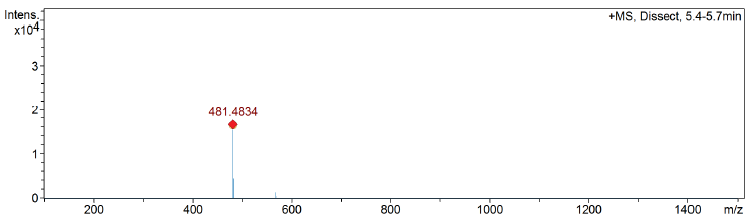


Figure 60S. MS^1^ spectrum of macrocyclic spermine based present in the ethanolic extract alkaloids of *A. niopoides* trunk


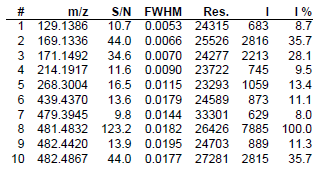

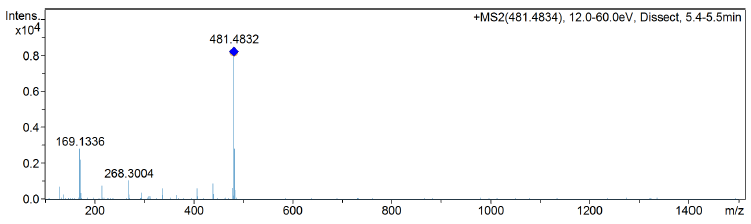


Figure 61S. MS^2^ spectrum of macrocyclic spermine based present in the ethanolic extract alkaloids of *A. niopoides* trunk

Figure 62S. Fragmentation pathways of macrocyclic spermine based (Compound 16).

**Compound (17) RT 5.6 min. MM 495 Da**

The compound follows the conserved fragmentation pathways established throughout the present study. The most informative product ions with **m/z 214** arise from cleavage of the aliphatic side chain together with the neutral loss of a C₂H₆N residue from the macrocyclic framework, yielding the characteristic diagnostic fragments of this class of compounds. The remaining fragmentation events are consistent with the previously described dissociation pathways and do not provide additional structural information beyond that already established (Figure 65S and 66S).


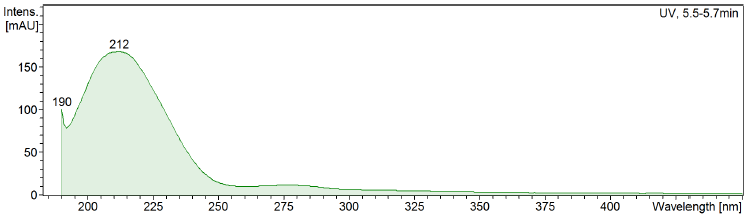


Figure 63S. UV spectrum of macrocyclic spermine based present in the ethanolic extract alkaloids of *A. niopoides* trunk


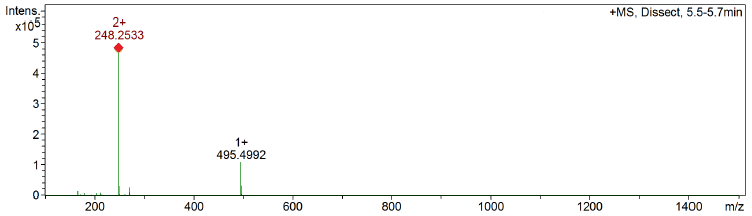


Figure 64S. MS^1^ spectrum of macrocyclic spermine based present in the ethanolic extract alkaloids of *A. niopoides* trunk


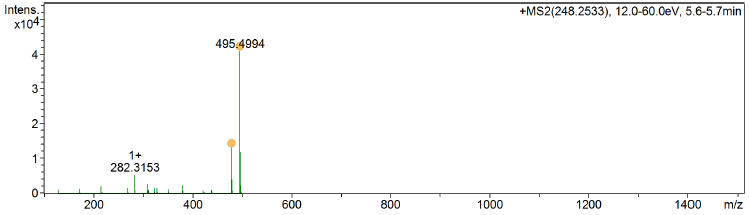


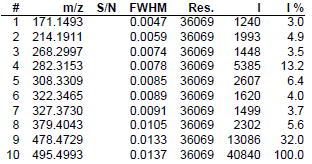


Figure 65S. MS^2^ spectrum of macrocyclic spermine based present in the ethanolic extract alkaloids of *A. niopoides* trunk

Figure 66S. Fragmentation pathways of macrocyclic spermine based (Compound 17).

**Compound (18) RT 5.8 min. MM 449 Da**


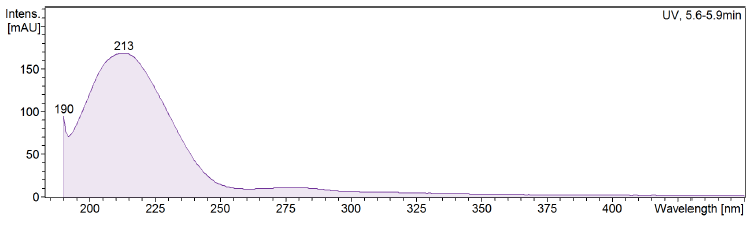


Figure 67S. UV spectrum of macrocyclic spermine based present in the ethanolic extract alkaloids of *A. niopoides* trunk


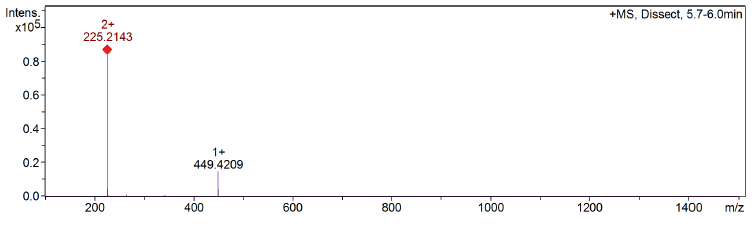


Figure 68S. MS^1^ spectrum of macrocyclic spermine based present in the ethanolic extract alkaloids of *A. niopoides* trunk


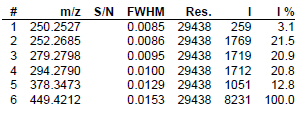

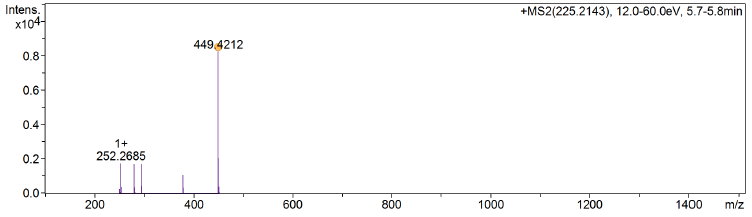


Figure 69S. MS^2^ spectrum of macrocyclic spermine based present in the ethanolic extract alkaloids of *A. niopoides* trunk

Figure 70S. Fragmentation pathways of macrocyclic spermine based (Compound 18).

**Compound (19) RT 5.9 min. MM 509 Da**

The compound follows the conserved fragmentation pathways established throughout the present study. The most informative product ions arise from cleavage of the aliphatic side chain, corresponding to the neutral loss of a C₁₈H₃₅ fragment, together with the characteristic elimination of a C₂H₆N residue from the macrocyclic framework. These dissociation pathways generate the diagnostic product ions that define this class of compounds. The remaining fragmentation events are consistent with the previously described fragmentation behavior and do not provide additional structural information or require further structural elucidation (Figure 73S and 74S).


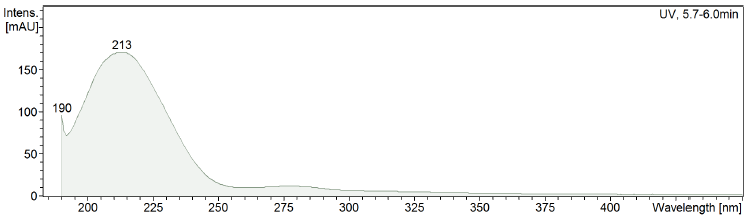


Figure 71S. UV spectrum of macrocyclic spermine based present in the ethanolic extract alkaloids of *A. niopoides* trunk


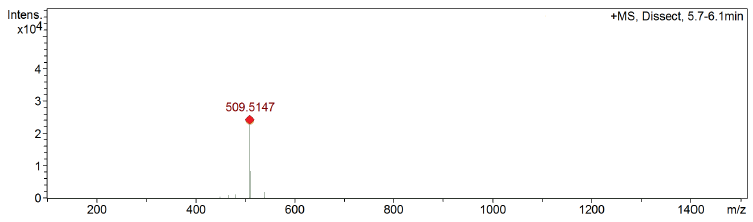


Figure 72S. MS^1^ spectrum of macrocyclic spermine based present in the ethanolic extract alkaloids of *A. niopoides* trunk


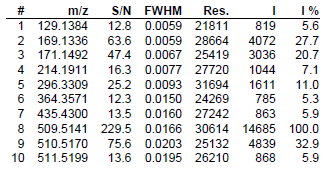

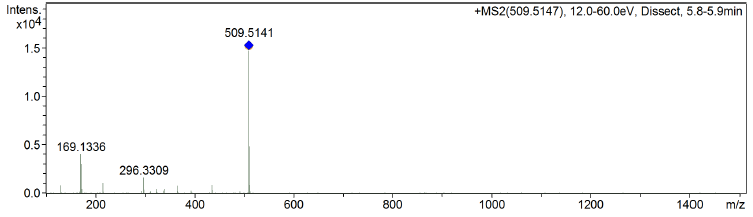


Figure 73S. MS^2^ spectrum of macrocyclic spermine based present in the ethanolic extract alkaloids of *A. niopoides* trunk

Figure 74S. Fragmentation pathways of macrocyclic spermine based (Compound 19).

**Compound (20) RT 6.0 min. MM 523 Da**

The compound follows the conserved fragmentation pathways established throughout the present study. The most informative product ions arise from cleavage of the aliphatic side chain, corresponding to the neutral loss of a C₁₉H₃₇ fragment, together with the characteristic elimination of a C₂H₆N residue from the macrocyclic framework. These fragmentation pathways generate the diagnostic product ions characteristic of this class of compounds. The remaining fragmentation events are consistent with the dissociation behavior described above and do not provide additional structural information beyond that already established (Figure 77S and 78S).

**
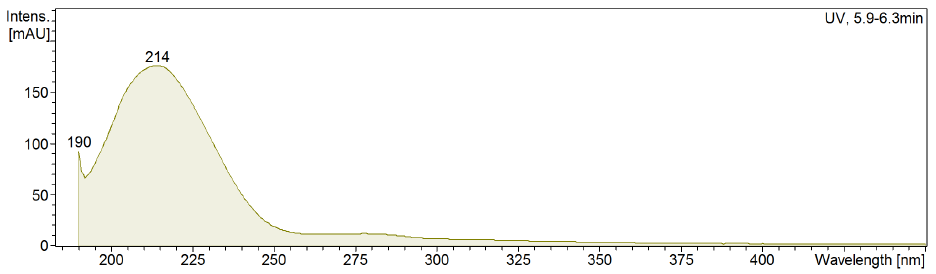
**

Figure 75S. UV spectrum of macrocyclic spermine based present in the ethanolic extract alkaloids of *A. niopoides* trunk

**
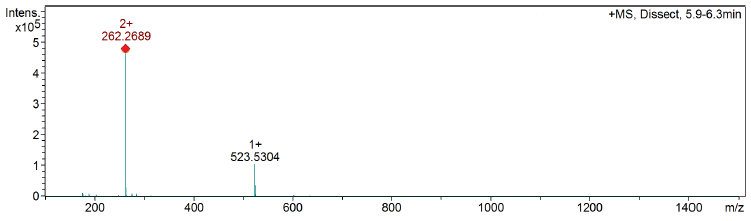

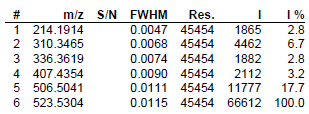
**

Figure 76S. MS^1^ spectrum of macrocyclic spermine based present in the ethanolic extract alkaloids of *A. niopoides* trunk

**
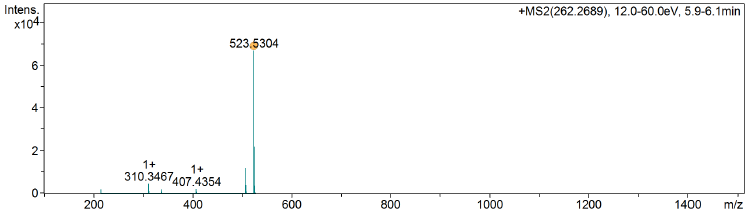
**

Figure 77S. MS^2^ spectrum of macrocyclic spermine based present in the ethanolic extract alkaloids of *A. niopoides* trunk

Figure 78S. Fragmentation pathways of macrocyclic spermine based (Compound 20).


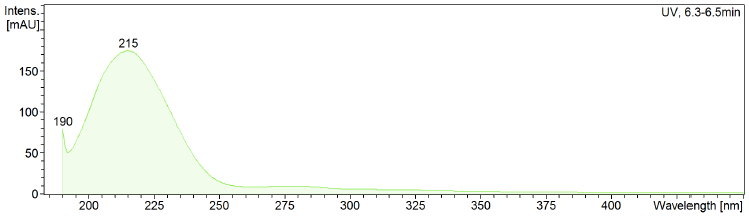
**Compound (21) RT 6.4 min. MM 551 Da**

Figure 79S. UV spectrum of macrocyclic spermine based present in the ethanolic extract alkaloids of *A. niopoides* trunk


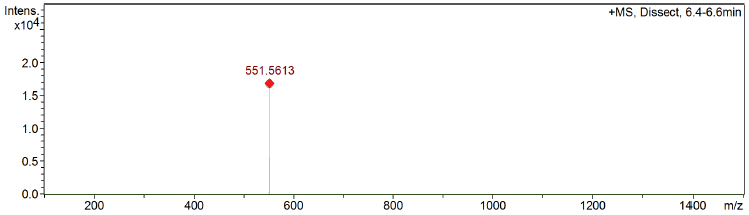


Figure 80S. MS^1^ spectrum of macrocyclic spermine based present in the ethanolic extract alkaloids of *A. niopoides* trunk


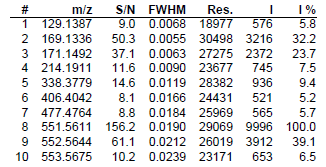

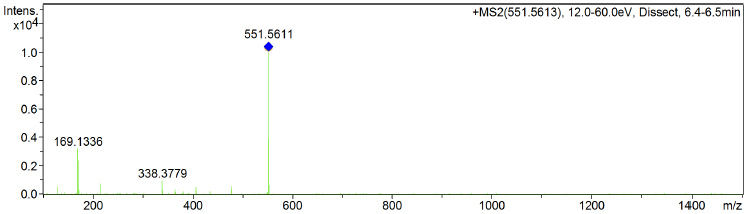


Figure 81S. MS^2^ spectrum of macrocyclic spermine based present in the ethanolic extract alkaloids of *A. niopoides* trunk

Figure 82S. Fragmentation pathways of macrocyclic spermine based (Compound 21).

**Compound (22) RT 6.5 min. MM 595 Da**

The fragmentation pathway is initiated by the neutral loss of carbon dioxide, yielding the product ion at m/z 551. This decarboxylation is consistent with the proposed presence of a geminal diol, resulting in the formation of a saturated side chain following CO₂ elimination. The subsequent fragmentation events follow the conserved dissociation pathways observed for structurally related compounds and do not provide additional information relevant to the structural assignment (Figure 85S and 86S).

**
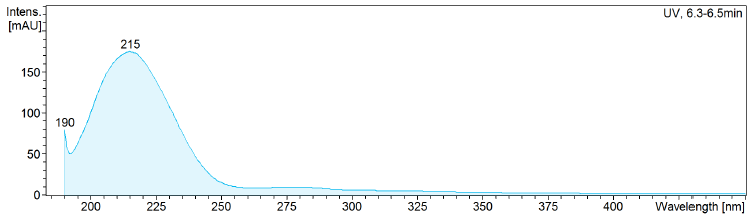
**

Figure 83S. UV spectrum of macrocyclic spermine based present in the ethanolic extract alkaloids of *A. niopoides* trunk

**
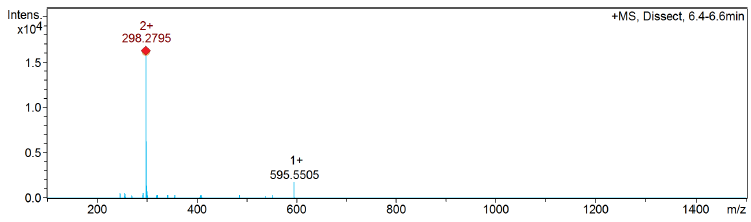
**

Figure 84S. MS^1^ spectrum of macrocyclic spermine based present in the ethanolic extract alkaloids of *A. niopoides* trunk

**
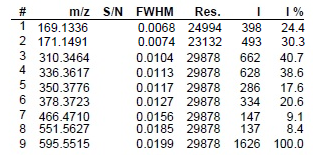

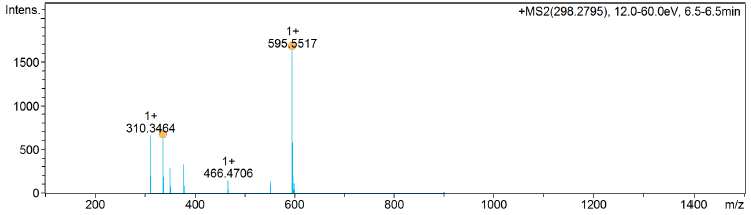
**

Figure 85S. MS^2^ spectrum of macrocyclic spermine based present in the ethanolic extract alkaloids of *A. niopoides* trunk

Figure 86S. Fragmentation pathways of macrocyclic spermine based (Compound 22).

**Compound (23) RT 6.6 min. MM 477 Da**

The observed fragmentation pattern is consistent with the conserved dissociation pathways described for the previously analyzed compounds. Among the most informative fragmentation events, the product ion at m/z 221 is generated from the m/z 393 precursor through an alkene-directed α-cleavage of the aliphatic side chain. This diagnostic fragmentation provides supporting evidence for the localization of one of the double bonds within the side chain. The remaining fragmentation events are consistent with the established fragmentation behavior of this class of compounds and do not contribute additional structural information (Figure 5 and 89S).


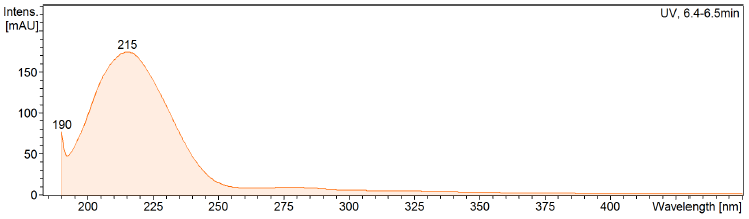


Figure 87S. UV spectrum of macrocyclic spermine based present in the ethanolic extract alkaloids of *A. niopoides* trunk


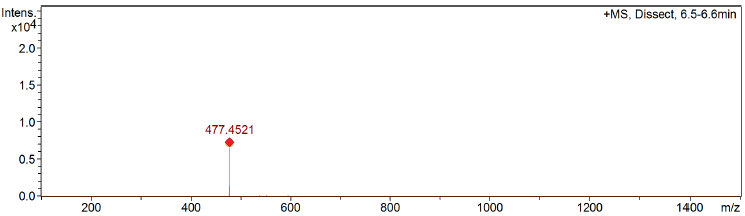


Figure 88S. MS^1^ spectrum of macrocyclic spermine based present in the ethanolic extract alkaloids of *A. niopoides* trunk


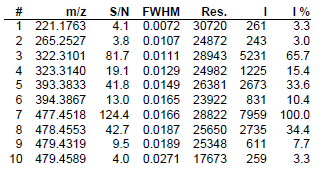

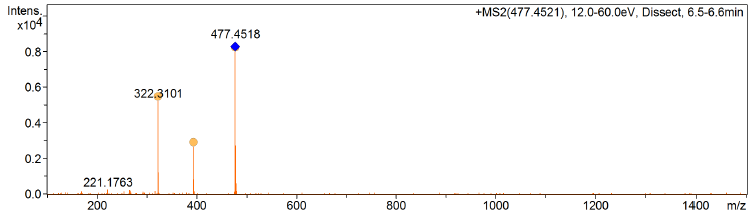


Figure 89S. MS^2^ spectrum of macrocyclic spermine based present in the ethanolic extract alkaloids of *A. niopoides* trunk

**Compound (24) RT 6.7 min. MM 505 Da**

The observed fragmentation pattern follows the conserved dissociation pathways described throughout the present study. A particularly informative fragmentation event involves the dissociation of the product ion at m/z 505, which undergoes cleavage of the macrocyclic framework concomitantly with α-cleavage adjacent to the terminal double bond of the aliphatic side chain. This process generates a diagnostic product ion that provides evidence for the position of the outermost double bond. The remaining fragmentation events are consistent with the characteristic fragmentation behavior of this class of compounds and do not contribute additional structural information (Figure 92S and 93S).


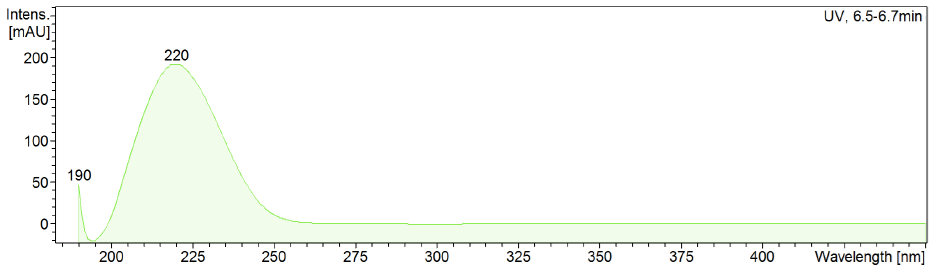


Figure 90S. UV spectrum of macrocyclic spermine based present in the ethanolic extract alkaloids of *A. niopoides* trunk


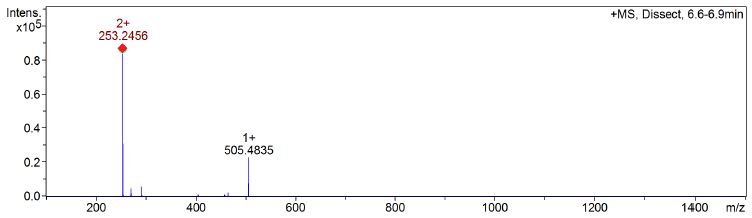


Figure 91S. MS^1^ spectrum of macrocyclic spermine based present in the ethanolic extract alkaloids of *A. niopoides* trunk


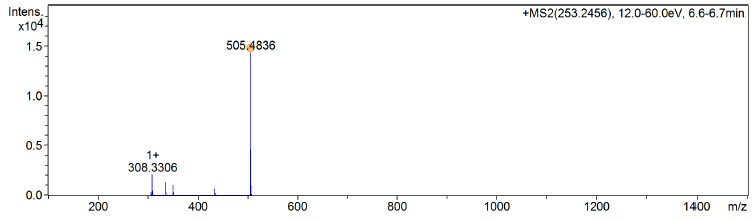


Figure 92S. MS^2^ spectrum of macrocyclic spermine based present in the ethanolic extract alkaloids of *A. niopoides* trunk

Figure 93S. Fragmentation pathways of macrocyclic spermine based (Compound 24).

**Compound (25) RT 7.1 min. MM 533 Da**

The observed fragmentation pattern follows the conserved dissociation pathways established for the analogous compounds investigated in the present study. Initial fragmentation of the protonated molecular ion at m/z 533 involves cleavage of the macrocyclic framework, yielding the product ion at m/z 429. Subsequent fragmentation of this ion is particularly informative, as it involves cleavage adjacent to the terminal double bond of the aliphatic side chain, resulting in the neutral loss of a 108 Da fragment. This diagnostic dissociation provides evidence for the position of one of the double bonds within the side chain, thereby supporting the proposed structural assignment

(Figure 96S and 97S).


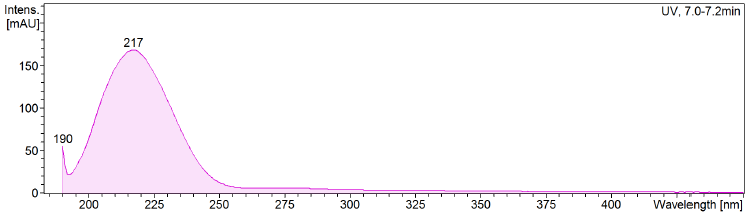


Figure 94S. UV spectrum of macrocyclic spermine based present in the ethanolic extract alkaloids of *A. niopoides* trunk

Figure 95S. MS^1^ spectrum of macrocyclic spermine based present in the ethanolic extract alkaloids of *A. niopoides* trunk


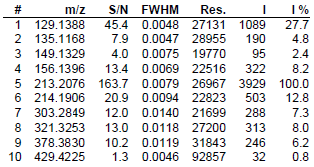

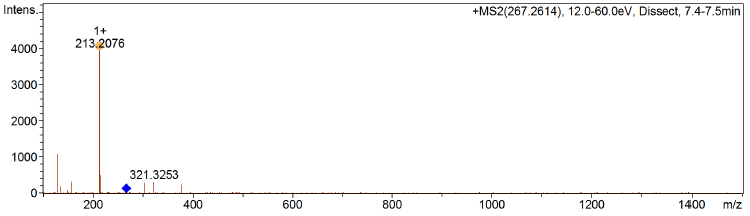

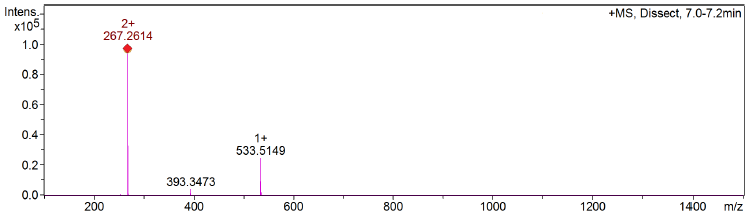


Figure 96S. MS^2^ spectrum of macrocyclic spermine based present in the ethanolic extract alkaloids of *A. niopoides* trunk

Figure 97S. Fragmentation pathways of macrocyclic spermine based (Compound 25).

**Compound (26) RT 8.9 min. MM 561 Da**

The ion at m/z 561 exhibited characteristic fragmentation pathways primarily involving cleavage of the polyamine macrocyclic moiety. The product ions observed at m/z 378 and m/z 379 were assigned to neutral losses of 183 Da (C₉H₁₇N₃O) and 182 Da (C₁₀H₁₈N₂O), respectively. These dissociation processes are consistent with C–N inductive cleavages. A further fragmentation pathway produced the low-mass ion at m/z 139 through the elimination of 240 Da (C₁₆H₃₂O) from the ion at m/z 379. In parallel, the ion at m/z 140 was generated directly from the precursor ion through the neutral loss of 421 Da (C₂₆H₅₁N₃O). The stability of this species can be **attributed** to the **stability** of **iminium ion** resonance. The **hydroxy** group position can **be** justified by the presence of the m/z **140** species. Additionally, the position of the double bond could be inferred from the loss of a C₂H₄ radical unit embedded within the neutral fragment eliminated from the macrocyclic moiety. This fragmentation is consistent with an α-cleavage process involving the cation radical generated at the olefinic site, leading to selective bond rupture adjacent to the original double-bond position. The resulting neutral loss provides diagnostic information regarding the location of unsaturation within the aliphatic chain and supports the structural assignment proposed for the compound (Figure 49S and 50S).


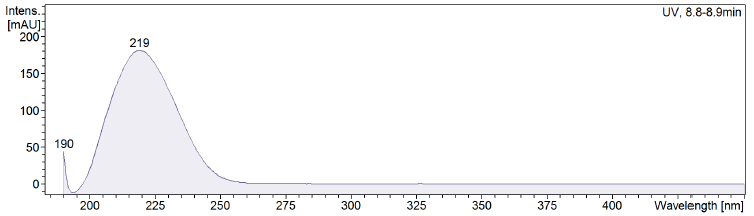


Figure 98S. UV spectrum of macrocyclic spermine based present in the ethanolic extract alkaloids of *A. niopoides* trunk

Figure 99S. MS^1^ spectrum of macrocyclic spermine based present in the ethanolic extract alkaloids of *A. niopoides* trunk

Figure 100S. MS^2^ spectrum of macrocyclic spermine based present in the ethanolic extract alkaloids of *A. niopoides* trunk

Figure 101S. Fragmentation pathways of macrocyclic spermine based (Compound 26).
